# Gene Expression Patterns for Proteins With Lectin Domains in Flax Stem Tissues Are Related to Deposition of Distinct Cell Wall Types

**DOI:** 10.3389/fpls.2021.634594

**Published:** 2021-04-26

**Authors:** Natalia Petrova, Alsu Nazipova, Oleg Gorshkov, Natalia Mokshina, Olga Patova, Tatyana Gorshkova

**Affiliations:** ^1^Laboratory of Plant Glycobiology, Kazan Institute of Biochemistry and Biophysics, FRC Kazan Scientific Center of RAS, Kazan, Russia; ^2^Laboratory of Plant Cell Growth Mechanisms, Kazan Institute of Biochemistry and Biophysics, FRC Kazan Scientific Center of RAS, Kazan, Russia; ^3^Institute of Physiology, FRC Komi Science Centre of Ural Branch of Russian Academy of Sciences, Syktyvkar, Russia

**Keywords:** plant lectins, flax, jacalin, malectin, amaranthin, gene expression, cell wall, gravitropic reaction

## Abstract

The genomes of higher plants encode a variety of proteins with lectin domains that are able to specifically recognize certain carbohydrates. Plants are enriched in a variety of potentially complementary glycans, many of which are located in the cell wall. We performed a genome-wide search for flax proteins with lectin domains and compared the expression of the encoding genes in different stem tissues that have distinct cell wall types with different sets of major polysaccharides. Over 400 genes encoding proteins with lectin domains that belong to different families were revealed in the flax genome; three quarters of these genes were expressed in stem tissues. Hierarchical clustering of the data for all expressed lectins grouped the analyzed samples according to their characteristic cell wall type. Most lectins differentially expressed in tissues with primary, secondary, and tertiary cell walls were predicted to localize at the plasma membrane or cell wall. These lectins were from different families and had various architectural types. Three out of four flax genes for proteins with jacalin-like domains were highly upregulated in bast fibers at the stage of tertiary cell wall deposition. The dynamic changes in transcript level of many genes for lectins from various families were detected in stem tissue over the course of gravitropic response induced by plant gravistimulation. The data obtained in this study indicate a large number of lectin-mediated events in plants and provide insight into the proteins that take part in tissue specialization and reaction to abiotic stress.

## Introduction

The term lectins joins a number of protein families capable of selectively recognizing various types of carbohydrates, while not interacting enzymatically with the recognized targets (Peumans and Van Damme, 1995). Lectins are widespread and present in all biological kingdoms (Sharon, 2008; Tsaneva and Van Damme, 2020). Initially, lectins were identified by the agglutination reaction; therefore, this group of proteins was often referred to as agglutinins. With the development of proteomics and genomics methods, the concept of lectins has moved to a fundamentally different qualitative level. It has been shown that not all lectins found during sequencing of plant genomes exhibit the property of agglutination (Van Damme et al., 2008).

The genomes of higher plants encode a variety of lectin proteins, which are classified into families according to their conserved carbohydrate-recognition domains (Van Damme et al., 2008; Jiang et al., 2010). Sequence bioinformatics studies across all available databases have shown that some of the lectin motifs are widespread (from plants to animals, fungi, and bacteria), while others are present only in certain plant families. Several informative papers with generalizations on plant lectin families have been published (Bellande et al., 2017; Van Holle et al., 2017; Tsaneva and Van Damme, 2020).

The absence of enzymatic interaction poses a number of unresolved questions about the functional role of lectins. As revealed by an inventory of plant lectins in several species with fully sequenced genomes, most plant proteins with lectin domains are multi-domain proteins in which one or more lectin domains are linked to other protein domains such as a protein kinase domain, an F-box domain, or a glycosyl hydrolase domain (Van Holle and Van Damme, 2019). The description of a plant protein as a lectin is often based only on the identification of a lectin motif in the encoding sequence; however, there is a lack of evidence on the ability to actually bind a carbohydrate, as well as for the characterization of the exact ligand specificity. Lectin specificity is often named due to a single monosaccharide, like mannose-binding or galactose-binding; it is clear though that lectins recognize more extended carbohydrate chains (Barre et al., 2019).

Proteins with lectin domains may have different types of architectures (Bellande et al., 2017). Lectin domains can be appended to intracellular kinase and trans-membrane domains to constitute receptor-like kinases; architecture of such proteins is designated as LecRLK type. Proteins with similar architecture but without kinase domains are designated LecRLP. Finally, the soluble proteins with neither kinase nor transmembrane domains are designated LecP. Plants are highly enriched in proteins with LecRLK architecture (Bellande et al., 2017). Proteins with lectin domains can be located in different cell compartments, including the vacuole, cytoplasm, nucleus, plasma membrane, and cell wall (Lannoo and Van Damme, 2010). Thus, the functions of different lectins may be diverse.

Some lectins accumulate in vacuoles as the defensive compounds against herbivorous animals and are quite abundant in seeds and vegetative storage tissues (Van Damme et al., 1998). However, in non-storage tissues, such as leaves, stems, roots, and flowers, the concentrations of lectins are low. Lectins were suggested to be involved in signaling reactions in plant cells or between plants and other organisms (Lannoo and Van Damme, 2010). The participation of lectins in a plant’s response to pathogens is supported by the numerous descriptions of the induced expression of lectins upon pathogen attack (Yan et al., 2005; Wakefield et al., 2006; Hwang and Hwang, 2011; Vandenborre et al., 2011). These inducible lectins are typically localized to the nucleus and/or cytoplasm of plant cells. Based on these observations, it was suggested that lectin-mediated protein-carbohydrate interactions in the cytoplasm and nucleus play an important role in the physiology of plant cell stress (Van Damme et al., 2004, 2008). In addition, lectins may also be involved in normal plant development, though the information on this topic is scarce. Several proteins with lectin domains were demonstrated to be involved in the regulation of plant growth and morphogenesis (Lee et al., 2003; Jiang et al., 2007; Duan et al., 2010). Research on plant lectins has mainly focused on the proteins of LecRLK type (Boisson-Dernier et al., 2011; Vaid et al., 2012; Bellande et al., 2017; He et al., 2018; Van Holle and Van Damme, 2018), since the presence of kinase domain allows for the direct coupling of these proteins with signaling pathways.

The bulk of plant polymeric carbohydrates are deposited in cell walls. The complex carbohydrate diversity in plant cell walls actively changes over the course of plant development, suggesting the presence of mechanisms capable of selectively recognizing the structural parameters of polysaccharides. Plants develop several types of cell walls that are based on different non-cellulosic polysaccharides. In dicots, primary cell walls are characterized by high proportions of xyloglucan and pectins (Carpita and Gibeaut, 1993), secondary cell walls are enriched with xylans (Zhong et al., 2019), and tertiary cell walls contain rhamnogalacturonans I with specific structure (Gorshkova et al., 2018a). The profiles of cell wall glycoproteins may also differ between the different types of cell walls, as suggested from proteomics (Nilsson et al., 2010; Bygdell et al., 2017) and transcriptomics studies (Gorshkov et al., 2018). Lectins that are secreted into the apoplast or are anchored to the plasma membrane have direct access to cell wall polysaccharides. If there are lectins specialized for the carbohydrate motifs of certain cell wall polymers, their expression could differ at deposition of various cell wall types.

To determine that we have used the developing flax (*Linum usitatissimum*) stem as an established model system that has tissues with three basic types of cell wall. Since proteomics is quite complicated for cell wall research and yields only a limited proportion of the expected proteins (Albenne et al., 2014), we conducted a transcriptomics analysis to compare the mRNA levels for all flax genes encoding proteins with lectin domains in various stem tissues of developing plants and during gravitropic response that exemplifies the effect of an abiotic stressor. The pronounced differential expression of lectins demonstrated the involvement of lectins in various aspects of plant physiology, and relation of some proteins with lectin domains to distinct cell wall types.

## Materials and Methods

### Input Data and Processing

**FIGURE 1:**
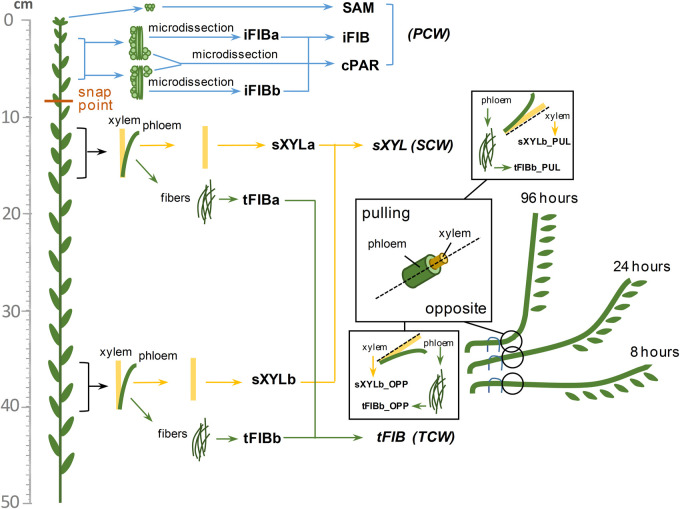
Diagram of the samples collected from the flax stem. Abbreviations: SAM, shoot apical meristem; cPAR, cortical parenchyma; iFIB, phloem fibers at the stage of intrusive elongation; sXYL, stem xylem with secondary cell wall; tFIB, phloem fibers with tertiary cell walls; PCW, primary cell wall; SCW, secondary cell wall, TCW, tertiary cell wall.

The 40 flax transcriptome libraries considered in this study were previously obtained by us and deposited in the Sequence Read Archive (SRA) as BioProjects (PRJNA475325, PRJNA631357). The four RNA-Seq datasets from the stem apex (PRJNA229810, Zhang and Deyholos, 2016) were downloaded from the European Nucleotide Archive^1^ as fastq-files of raw data. The listed datasets include samples from various tissues of the flax stem consisting of cells with different types of cell wall. The detailed localization of samples on the flax stem is shown in Figure 1. The SAM (shoot apical meristem) and cPAR (cortical parenchyma) samples consist of cells with primary cell wall (PCW), same as the iFIBa and iFIBb samples that contain phloem fibers at the stage of intrusive growth; iFIBa, iFIBb and cPAR samples were isolated by cryosectioning and laser microdissection (Gorshkova et al., 2018b). The samples of xylem stem part (sXYLa, sXYLb) were enriched in cells with secondary cell walls (SCW) and isolated phloem fibers at the stage of tertiary cell wall (TCW) deposition constituted the samples tFIBa and tFIBb (Figure 1). Additionally, the set of SCW and TCW samples was obtained from the region of stem curvature formed in the course of gravitropic response to return the inclined plants to vertical position (Ibragimova et al., 2017; Gorshkov et al., 2018). Each segment of gravibending stems was cut along into halves and separated into pulling (PUL) and opposite (OPP) sides both in the xylem (inner part) and in the phloem (outer part); the outer peel of each stem side was used to isolate phloem fibers. Segments of the flax stem were collected 8 h, 24h and 96h after plant inclination (Figure 1).

After processing of raw reads by the BBDuk utility of BBTools v 37.02^2^ the clean reads were mapped onto the flax genome downloaded from Phytozome v 12 (Goodstein et al., 2012; Wang et al., 2012) using HISAT2 v2.1.083 (Kim et al., 2015). Transcript abundance was determined by StringTie v2.0 (Pertea et al., 2016) and the number of all reads for each gene were calculated as total gene read (TGR) counts. DESeq2 v.1.28.1 as R package was used to normalize the counts per gene by the estimateSizeFactors function and to perform pairwise differential expression analysis (Love et al., 2015).

### Identification of Genes Encoding Lectins of Different Families

The plant genes for proteins with lectin domains of different families were recognized by the name search of characteristic pfam domains (Pfam 33.1 database^3^; El-Gebali et al., 2019) in the Phytozome v12.1.6 database (^4^ Goodstein et al., 2012). The pfam domain names that were used to identify genes are listed in Table 1. Members of CRA lectin family were identified by the presence of cd02879 (GH18_plant_chitinase_class_V) domain using the CD-search tool (^5^ Lu et al., 2020). The protein sequences of plant genes were downloaded from the Phytozome v12.1.6 and from the Uniprot (release 2020_05) (^6^ The UniProt Consortium, 2019) databases. For each protein with an unreliable amino acid sequence (truncated, or significantly different from orthologous genes, or including not typical domains that were not in other members, etc.) based on genome sequence from Phytozome, the open reading frame as well as the intron-exon structure and protein sequence were re-predicted using Augustus program^7^, some sequences were additionally checked by FGENESH (^8^ Solovyev et al., 2006); if for any reason a reliable sequence for such proteins was not established (errors or gaps in the sequencing, SNPs, etc.) the sequence was used as taken from Phytozome. Revised sequences are presented in Supplementary File 1.

**TABLE 1:** Distribution of proteins with lectin domains in the flax genome by families.

**Family (according to Phytozome)**	**Tsaneva and Van Damme, 2020**	**Jiang et al., 2010**	**Designation used in this article**	**Pfam ID**	**Number of family members in the flax genome**	**Number of family members expressed in flax stem**	**Number of family members with SP**	Number of family members with TM/*	**Description (according to Phytozome)**
B_lectin	GNA	B_lectin	GNA	PF01453	112	88	104	97/87	D-mannose binding lectin
Lectin_legB	Legume lectin domain	Lectin_legB	Legume	PF00139	72	52	63	61/58	Legume lectin domain
Malectin	-	-	Malectin	PF11721	39	37	32	33/32	Di-glucose binding within endoplasmic reticulum
PP2	Nictaba	Phloem	Nictaba	PF14299	37	33	0	0	Phloem protein 2
LysM	LysM domain	LysM	LysM	PF01476	36	21	29	25/18	LysM domain
Gal_Lectin	-	Gal_lectin	Galactose-binding lectin	PF02140	23	15	17	8/0	Galactose binding lectin domain
Malectin_like	-	-	Malectin-like	PF12819	22	18	17	17/15	Carbohydrate-binding protein of the ER
Agglutinin	Amaranthin domain	-	Amaranthin	PF07468	19	7	0	0	Agglutinin domain
Chitin_bind_1	Hevein domain	Chitin_bind_1	Hevein	PF00187	17	4	16	8/0	Chitin recognition protein (Hevein)
Gal-bind_lectin	-	Gal_binding_Lectin	Galectin-like	PF00337	11	11	0	11/11	Galactoside-binding lectin (galectin)
Calreticulin	-	Calreticulin	Calreticulin	PF00262	8	8	8	5/2	Calreticulin family
Jacalin	Jacalin-related domain	Jacalin	Jacalin	PF01419	4	4	0	0	Jacalin-like lectin domain
CRA	CRA	-	CRA	-	4	1	4	4/0	class V chitinase-related agglutinin
Lectin_C	-	Lectin_C	C-type	PF00059	2	2	2	2/2	Lectin C-type domain
EUL	EUL domain	EEA	EUL	PF14200	1	1	0	0	Ricin-type beta-trefoil lectin domain-like
**Total**					**407**	**302**	**292**	**271/225**	

Families are listed according to the number of members in the flax genome in the descending order. Abbreviations: SP – signal peptide (the prediction was performed using SignalP-5.0 (http://www.cbs.dtu.dk/services/SignalP/; Armenteros et al., 2019), PrediSi (http://www.predisi.de/home.html), iPSORT (http://ipsort.hgc.jp/index.html; Bannai et al., 2002), TM – transmembrane domain (the prediction was performed using TMHMM v2.0 (http://www.cbs.dtu.dk/services/TMHMM/; Krogh et al., 2001), *– the number of family members with TM, except those with only one N-terminal TM, which according to TMHMM could be a possible signal sequence (signal peptide for this sequences was also predicted by SignalP).

The prediction of a signal peptide was performed using number of bioinformatics resources: SignalP-5.0 (^9^ Armenteros et al., 2019), PrediSi^10^, iPSORT (^11^ Bannai et al., 2002). The presence of transmembrane domain was performed using TMHMM v2.0 (^12^ Krogh et al., 2001) (Supplementary File 1). Domain organization of flax members of lectin families were resolved using the InterProScan tool implemented in the InterPro database (^13^ Mitchell et al., 2019) and was visualized in Adobe Illustrator CC 2017 software.

The putative subcellular localization of flax lectins was revealed as predicted for their *A. thaliana* homologs using SUBA4 (^14^ Hooper et al., 2017) and using LocTree3 service (^15^ Goldberg et al., 2014).

### Quantification and Tissue Specificity Analysis of Gene Expression

In total, 32,870 from 43,486 genes were considered as expressed according to the cut-off TGR ≥ 16 at least in one sample (SEQC/MAQC-III Consortium, 2014) and were used for the analysis of differential expression. The regularized-logarithm transformation or rlog (Love et al., 2015) of the raw count data was used for hierarchical clustering of genes encoding proteins with lectins domain and the visualization by heatmap using the hclust function in R (R Core Team, 2014). A dendrogram and a heatmap were generated by the R function heatmap.2.

To characterize the differential expression of lectin genes depending on the type of cell wall at the input to DESeq2 we used datasets designated as PCW, SCW, TCW. In particular, dataset of PCW samples consisted of SAM, cPAR, iFIBa and iFIBb samples as biological replicates, SCW set consisted of sXYLa and sXYLb, whereas the tFIBa and tFIBb samples were used as biological replicates for TCW dataset. To be considered as differentially expressed, genes identified by DESeq2 were required to have at least the 2-fold change and padj < 0.01. The data of RNA-Seq were verified by qRT-PCR analysis for the selected 10 genes (Supplementary File 2).

To analyze the changes in gene expression in the course of graviresponse, the rlog-dataset of samples obtained from gravistimulated stems was used as input for clustering based not on absolute expression value but rather on the amount by which each gene deviates in a specific sample from the gene’s average across all samples (Love et al., 2015). Hence, in this case we centered the values of each gene in the samples and built a heatmap where color corresponds to the amount by which a gene expression variance deviates from the gene’s mean variance across all samples.

Additionally, on the basis of normalized TGR values for the lectin genes we calculated the index tau as tissue specificity score of a gene (Yanai et al., 2005; Kryuchkova-Mostacci and Robinson-Rechavi, 2016) using the roonysgalbi/tispec package in R^16^. The total set of samples to calculate the index tau included SAM, cPAR, the combined sample of iFIB as an average of iFIBa and iFIBb; sXYLa and sXYLb were averaged with the name sXYL, whereas tFIB was represented by averaging values of expression in tFIBa and tFIBb from plants grown in normal condition (Figure 1).

### Phylogenetic Analysis

The obtained sequences of plant lectin-domain containing proteins were subjected to multiple alignments using the web-based service ClustalW (^17^ Madeira et al., 2019). The alignments were further exposed to a maximum likelihood phylogenetic analysis in IQTREE1.6.9 software (Nguyen et al., 2015). The best-fit models of sequence evolution were automatically computed in ModelFinder (IQTREE1.6.9) (Kalyaanamoorthy et al., 2017). Candidate models were selected according to Bayesian Information Criterion (BIC). The ultrafast bootstrap branch support (Minh et al., 2013) with 10,000 replicates was used to construct each dendrogram (values less than 95 are not significant). Unrooted trees were visualized using the web-based service iTOL 5.3 (^18^ Letunic and Bork, 2019) and corrected in Adobe Illustrator CC 2017.

## Results

### Lectin Genes in the Flax Genome

The search for genes encoding lectin domain-containing proteins was performed using the Pfam database^19^ and the lists of lectin domains present in literature (Van Damme et al., 2008; Jiang et al., 2010; Bellande et al., 2017; Van Holle and Van Damme, 2019). It allowed for the identification of 407 genes in the flax genome (Table 1), which is approximately 1% of the total number of genes in the flax genome. We found representatives of 15 out of 18 described families of plant proteins with lectin domains (Table 1). Genes for members of 11 classical lectin families were recognized: amaranthins, calreticulins, homologs of class V chitinases (CRA), C-type lectins that require calcium ions for carbohydrate binding, the *Euonymus europaeus* lectin (EUL) family, the *Galanthus nivalis* agglutinin (GNA) family, the hevein family, the jacalin-related lectin family, the legume lectin family, the lysin motif (LysM) family, and the *Nicotiana tabacum* agglutinin (Nictaba) family. Lectins of the *Agaricus bisporus* agglutinin (ABA) and Cyanovirin-N (CV-N) families were not detected and have not been previously identified in angiosperms (Van Holle and Van Damme, 2019). Genes for members of the ricin B lectin family were not reliably identified in the current version of the flax genome, though they are present in other higher plants (Van Holle and Van Damme, 2019).

Representatives of two protein families, malectin and malectin-like, that are often, but not always considered among plant proteins with lectin domains (Bellande et al., 2017) were also added to the list (Table 1). Malectin and malectin-like proteins are included in the CAZy database (^20^ Henrissat and Davies, 1997) because in bacteria, malectin domains (PF11721 and structurally similar PF12819) are attached to various glycosidase domains (GH2, GH16). However, in plant proteins, malectin and malectin-like domains are not combined with the domains that are enzymatically active on carbohydrates (Bellande et al., 2017; Franck et al., 2018).

Two other protein families, which in addition to a lectin domain have another domain that indicates enzymatic activity with respect to the bound carbohydrate, were included in this study. Galactose-binding lectin and galectin-like proteins have domains characteristic for β-galactosidase and for galactosyltransferase, respectively. These proteins are present in the classification of plant lectins by Jiang et al. (2010), but are absent in the lists of lectins given by other researchers (Van Damme et al., 2008; Bellande et al., 2017). Based on the presence of the domains characteristic for lectins, we included these groups of proteins in our study. In the flax genome, galactose-binding lectins and galectin-like families contained 23 and 11 genes, respectively (Table 1).

Lectin families may have different names in the literature. For example, the GNA-lectins (*Galanthus nivalis* agglutinin; named according to the plant from which the first representative with a similar domain in plants was isolated) (Van Damme et al., 1987) are also named B-type lectins (Jiang et al., 2010), D-mannose-binding lectins [Phytozome v12.1.6 database ((See text footnote 4)Goodstein et al., 2012)], and G-type lectins (Bellande et al., 2017). Many LecRLKs belonging to this family are also known as S-locus protein kinases due to the presence of the S-locus domain, which is involved in pollen self-incompatibility (Van Holle et al., 2017). All abbreviations of lectin family names used in this text are given in Table 1.

Each lectin sequence was analyzed for the presence of a signal peptide and transmembrane domains. Members of several lectin families, including Nictaba, amaranthin, jacalin, and EUL, have neither signal nor transmembrane peptides (Table 1, Supplementary File 1). Such proteins are translated on the free ribosomes in the cytoplasm and later remain in the cytoplasm or can be translocated into the nucleus (Lannoo and Van Damme, 2010). Signal peptides were detected in the vast majority of the proteins from most of the lectin families, suggesting that they are synthesized on the ribosomes attached to the endoplasmic reticulum and can be retained in this compartment or further transported through the Golgi apparatus, exposed to the extracellular space, anchored to the plasma membrane, or deposited into the cell wall; another possibility is transportation to the vacuoles. Altogether, two thirds of flax lectins are membrane-bound, as indicated by the presence of transmembrane domains (Table 1).

### General Characteristics of Lectin Expression in Flax Stem Tissues

To analyze the expression of genes for lectins in different parts of the flax stem, previously published RNA-Seq data (Zhang and Deyholos, 2016; Gorshkov et al., 2018, 2019; Gorshkova et al., 2018b; Mokshina et al., 2020) were used. The shoot apical meristem (SAM) sample contains young cells of various stem tissues at the beginning of their development. Cell division and elongation coupled with the formation of the primary cell wall (PCW) are characteristic for this stem zone. Primary cell wall deposition is also characteristic for fibers at the stage of intrusive elongation (iFIB) and for young cortical parenchyma (cPAR). Several cell types at a considerably more advanced stage of development constituted sXYL samples. Vessels, xylem fibers, and parenchyma of these samples have secondary cell walls (SCW) and are mainly involved in water transport and plant mechanical support (Esau, 1965). Finally, tFIB samples consist of the only cell type at a certain stage of development – phloem fibers that deposit tertiary cell walls (TCW) of specific composition, architecture, and function (Gorshkova et al., 2018a).

The expression levels of all lectin genes in the analyzed stem tissues of developing flax plants are given in Supplementary File 3. Approximately 75% of the genes for proteins with lectin domains present in the flax genome and constituting the families listed in Table 1 were expressed in at least one analyzed stem sample (threshold of TGR value ≥ 16).

**FIGURE 2:**
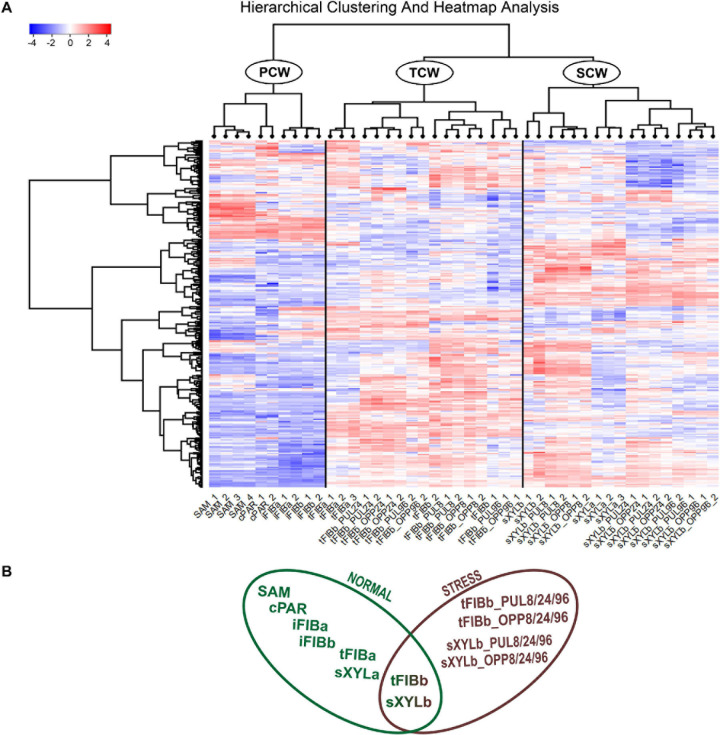
Expression pattern of genes encoding proteins with lectin domains in all of the analyzed tissue samples collected from a flax stem **(A)** and datasets used in the analyses **(B)**. A heatmap with a dendrogram of hierarchical clustering is displayed in a grid where each row represents a gene (*n* = 302) and each column represents a sample (*n* = 44) with replicates. The color scale (–4 to 4) represents the Z–score, calculated by normalized expression values for lectin genes in different tissues (TGR ≥ 16 in at least one sample), where blue-white-red represents the genes with down–, unchanged, and upregulated expression, respectively. PCW, SCW, and TCW are the major clusters of samples (see text). Abbreviations: PCW, primary cell wall; SCW, secondary cell wall; and TCW, tertiary cell wall; tFIBb and sXYLb, isolated fibers with TCW and stem xylem with SCW of non-inclined plant, respectively; tFIBb_PUL8, tFIBb_PUL24, tFIBb_PUL96 – isolated fibers with TCW from pulling side of flax stem after 8, 24, 96 h of gravibending, respectively; tFIBb_OPP8, tFIBb_OPP24, tFIBb_OPP96 – isolated fibers with TCW from opposite side of flax stem after 8, 24, 96 h of gravibending, respectively; sXYLb_PUL8, sXYlb_PUL24, sXYLb_PUL96 – pulling side of stem xylem with SCW after 8, 24, 96 h of gravibending, respectively; sXYLb_OPP8, sXYlb_OPP24, sXYLb_OPP96 – opposite side of stem xylem with SCW after 8, 24, 96 h of gravibending.

To better understand the variation of lectin gene expression across all samples, including stem tissues of plants grown under normal conditions and after plant inclination and development of gravitropic response, we performed the hierarchical clustering of 302 expressed genes (TGR ≥ 16 in at least one sample) encoding lectins of various families. According to the resulting heatmap (Figure 2A) all samples were subdivided into three clusters that corresponded to the cell wall type. The PCW cluster that contained SAM, cPAR, and iFIB samples (*n* = 10) consisting of cells with primary cell wall, was separated from SCW and TCW clusters. The SCW and TCW clusters were closer in distance to each other than to PCW, and formed two separate clusters. Cluster SCW contained xylem tissues (*n* = 17) that included mainly cells with secondary walls, whereas the TCW cluster consisted of samples represented by phloem fibers at the stage of tertiary cell wall deposition (*n* = 17).

Collectively, the visualization of expression patterns by heatmap demonstrated the presence of gene expression signatures associated with the distinct cell wall type both within the course of plant development and during gravitropic response induced by stem inclination. The findings herein determined the downstream analyses, which were conducted using two datasets: flax stem tissues under normal growth conditions and under conditions inducing a gravitropic response (Figure 2B). Further we considered the representatives of lectin families with certain expression characteristics starting from the dataset for samples collected from plants grown under normal conditions.

### Genes for Proteins With Lectin Domains That Have Stable Expression Levels in All Analyzed Flax Tissues

Some genes encoding proteins with lectin domains exhibited similar levels of mRNA abundance in all analyzed stem parts. To identify the pool of stably expressed lectin genes, we calculated the tau-score for each lectin transcript in all samples of the dataset from the stem tissues under normal growth conditions (Figure 2B; Supplementary File 3). The tau-score may range from 0 to 1, with 0 and 1 indicating ubiquitously and specifically expressed genes, respectively (Yanai et al., 2005; Kryuchkova-Mostacci and Robinson-Rechavi, 2016). Based on the observed tau-scores (Supplementary File 3), the pool of flax lectin genes was extracted with the scores less than 0.15 that indicated an expression profile similar to house-keeping genes (Table 2).

**TABLE 2:** *Linum usitatissimum* genes encoding proteins with lectin domains with relatively constant expression values (the mean values of tau-score < 0.15 across all samples).

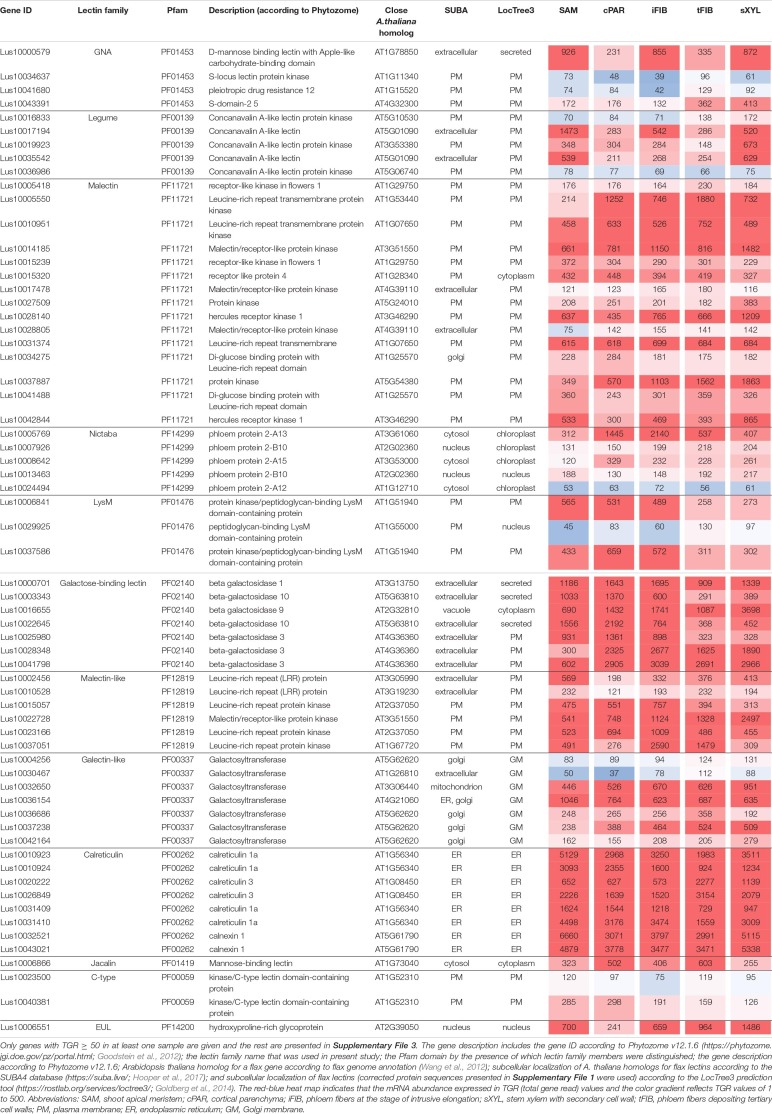

*Only genes with TGR ≥ 50 in at least one sample are given and the rest are presented in Supplementary File 3. The gene description includes the gene ID according to Phytozome v12.1.6 (https://phytozome.jgi.doe.gov/pz/portal.html; Goodstein et al., 2012); the lectin family name that was used in present study; the Pfam domain by the presence of which lectin family members were distinguished; the gene description according to Phytozome v12.1.6; *Arabidopsis thaliana* homolog for a flax gene according to flax genome annotation (Wang et al., 2012); subcellular localization of *A. thaliana* homologs for flax lectins according to the SUBA4 database (https://suba.live/; Hooper et al., 2017); and subcellular localization of flax lectins (corrected protein sequences presented in Supplementary File 1 were used) according to the LocTree3 prediction tool (https://rostlab.org/services/loctree3/; Goldberg et al., 2014). The red-blue heat map indicates that the mRNA abundance expressed in TGR (total gene read) values and the color gradient reflects TGR values of 1 to 500. Abbreviations: SAM, shoot apical meristem; cPAR, cortical parenchyma; iFIB, phloem fibers at the stage of intrusive elongation; sXYL, stem xylem with secondary cell wall; tFIB, phloem fibers depositing tertiary cell walls; PM, plasma membrane; ER, endoplasmic reticulum; GM, Golgi membrane.*

For the calreticulin (PF00262) family, high gene expression levels were found for all eight members in all analyzed tissues (Table 2), belonging to putative calreticulins (CALR - *Lus10010923*, *Lus10010924*, *Lusl0031409*, *Lus10031410*; CALRETICULIN-3 – *Lus10020222*, *Lus10026849*) and calnexins (CANX – *Lus10032521*, *Lus10043021*). The *A. thaliana* homologs of these genes encode glucose-binding lectins located in the endoplasmic reticulum (Lannoo and Van Damme, 2014). All flax lectins from the calreticulin family were predicted by the LocTree3 program to be localized in the endoplasmic reticulum (Table 2).

**FIGURE 3:**
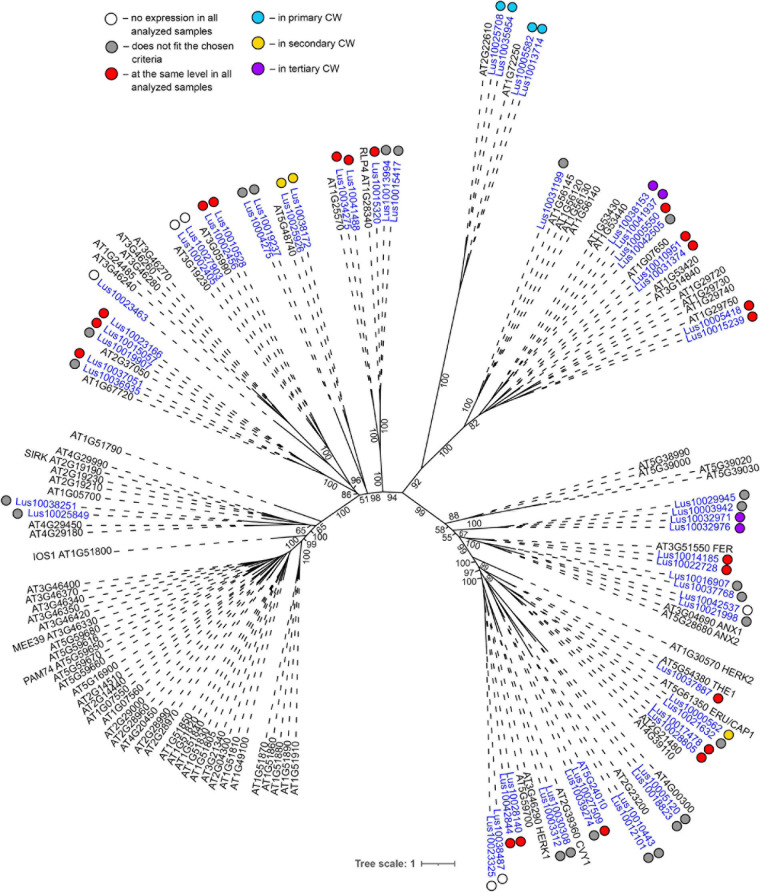
Phylogenetic dendrogram of malectin (PF11721) and malectin–like (PF12819) family members of *Arabidopsis thaliana* and *Linum usitatissimum*. The *A. thaliana* gene names are given in black font; additional gene names are given according to descriptions in the Uniprot database (https://www.uniprot.org/; The UniProt Consortium, 2019). The *L. usitatissimum* gene names are given in dark blue font. The different colored dots next to *L. usitatissimum* gene names indicate genes with different expression patterns. The criteria used for different expression pattern identification as follow: red, the mean values of tau-score < 0.15 across all samples; light blue, DEGs upregulated in samples with primary CW; yellow, DEGs upregulated in samples with secondary CW; purple dots, DEGs upregulated in samples with tertiary CW. Flax genes with red, light blue, yellow, and purple dots are listed in Tables 2–5, respectively. White dots indicate genes that are not expressed in the analyzed flax samples, and gray dots indicate genes where the expression does not fit the chosen criteria for a certain expression pattern (expression values for these genes are given in Supplementary File 3). Numbers indicate the ultrafast bootstrap support values for some branches. Abbreviations: DEGs, differentially expressed genes, a pairwise comparison log_2_FC ≥ 1; padj ≥ 0.01, CW, cell wall.

Genes for plasma membrane-localized proteins with lectin domains that had similar expression levels in all analyzed tissues included a noticeable proportion of genes with malectin (PF11721) and structurally related malectin-like (PF12819) domains (Table 2). Out of 61 flax genes that contain PF11721 or PF12819, one third was expressed at relatively constant levels (Figure 3, red dots). Most of these flax genes had LecRLK architecture, including Lus10028140 and Lus10042844, which are both homologous to AT3G46290 for HERKULES1 (HERK1) reported to take part in the regulation of plant development (Li et al., 2016). Lus10037887 (Figure 3) is homologous to AT5G54380 for THESEUS1 (THE1), a receptor kinase that mediates the response of growing plant cells to the perturbation of cellulose synthesis and may act as a cell-wall-integrity sensor (Hématy et al., 2007; Guo et al., 2009). In addition, constant expression levels (Table 2) were detected for genes encoding transmembrane proteins that have malectin domains but no kinase domains, like Lus10015320, Lus10034275, and Lus10041488; these were grouped together with RLP4 (AT1G28340) on the phylogenetic tree (Figure 3).

**FIGURE 4:**
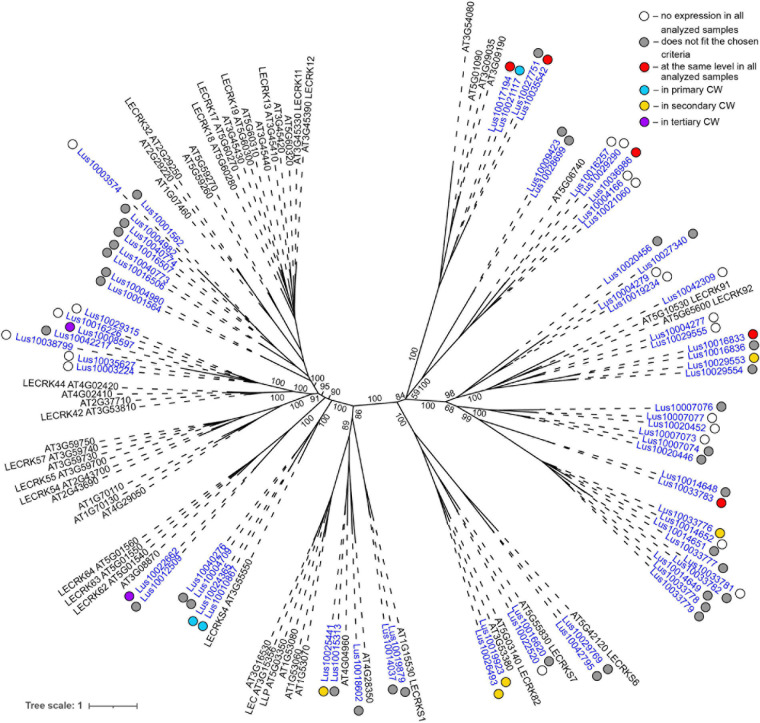
Phylogenetic dendrogram of legume (PF00139) lectin family members of *Arabidopsis thaliana* and *Linum usitatissimum*. The *A. thaliana* gene names are given in black font; additional gene names are given according to descriptions in the Uniprot database (https://www.uniprot.org/; The UniProt Consortium, 2019). The *L. usitatissimum* gene names are given in dark blue font. The different colored dots next to *L. usitatissimum* gene names indicate genes with different expression patterns. The criteria used for different expression pattern identification as follow: red, the mean values of tau-score < 0.15 across all samples; light blue, DEGs upregulated in samples with primary CW; yellow, DEGs upregulated in samples with secondary CW; purple dots, DEGs upregulated in samples with tertiary CW. Flax genes with red, light blue, yellow, and purple dots are listed in Tables 2–5, respectively. White dots indicate genes that are not expressed in the analyzed flax samples, and gray dots indicate genes where the expression does not fit the chosen criteria for a certain expression pattern (expression values for these genes are given in Supplementary File 3). Numbers indicate the ultrafast bootstrap support values for some branches. Abbreviations: DEGs, differentially expressed genes, a pairwise comparison log_2_FC ≥ 1; padj ≥ 0.01, CW, cell wall.

Low level of expression specificity between various stem tissues of flax was also detected for the genes encoding plasma membrane-localized proteins from several other lectin families, like legume lectins, GNA lectins, C-type, and LysM (Table 2). C-type (PF00059) lectins are scarce in plant genomes, but are widely present in vertebrates (Tsaneva and Van Damme, 2020); flax has only two C-type lectins (Table 1) and both were expressed in all analyzed stem tissues (Table 2). Legume lectins (PF00139) (such as concanavalin A from jack beans (Leguminosae family), found in abundance in seeds of this taxonomic group) are quite numerous in the flax genome (Figure 4), which is similar to findings in other plant species (Bellande et al., 2017; Van Holle et al., 2017). Representatives of legume lectins have very divergent expression patterns (Figure 4, dots of different colors).

Low tau scores were also found for several genes for proteins with PF14299 (Table 2). According to Phytozome, this domain is named PP2 (phloem protein 2); however, its carbohydrate-recognizing part is designated as Nictaba sequence (Van Holle and Van Damme, 2018). Within the framework of used classification, proteins with the PP2 domain belong to the Nictaba lectin family. The Nictaba domain specifically recognizes high-mannose *N*-glycans, complex *N*-glycans, and, to a lesser extent, – GlcNAc oligomers (Tsaneva and Van Damme, 2020). Proteins with a Nictaba domain are considered nucleocytoplasmic and were confirmed to interact with O-GlcNAc-modified histones in the nucleus (Delporte et al., 2014).

**FIGURE 5:**
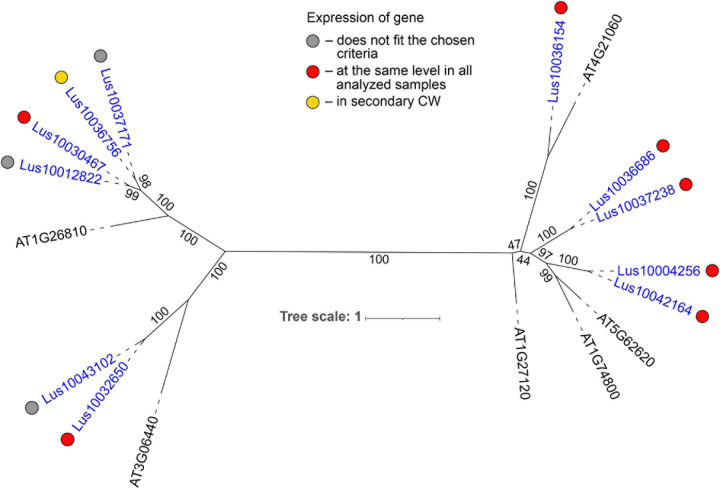
Phylogenetic dendrogram of galectin–like (PF00337) domain-containing glycosyltransferases from family 31 of *Arabidopsis thaliana* and *Linum usitatissimum*. The *A. thaliana* gene names are given in black font; additional gene names are given according to descriptions in the Uniprot database (https://www.uniprot.org/; The UniProt Consortium, 2019). *L. usitatissimum* gene names are given in dark blue font. The numbers in parentheses reflect the number of predicted transmembrane domains according to TMHMM v2.0 prediction tool (http://www.cbs.dtu.dk/services/TMHMM/; Krogh et al., 2001). The different colored dots next to *L. usitatissimum* gene names indicate genes with different expression patterns. The criteria used for different expression pattern identification as follow: red, the mean values of tau-score < 0.15 across all samples; yellow, DEGs upregulated in samples with secondary CW. Flax genes with red and yellow dots are listed in Tables 2, 4, respectively. Gray dots indicate genes where the expression does not fit the chosen criteria (tau-score < 0.15 or DEGs) for a certain expression pattern (expression values for these genes are given in Supplementary File 3). Numbers indicate the ultrafast bootstrap support values for some branches. Abbreviations: DEGs – differentially expressed genes, a pairwise comparison log_2_FC ≥ 1, padj ≥ 0.01, CW, cell wall.

Most of the flax genes (e.g., *Lus10032650*, *Lus10037238*, and *Lus10036154*) for proteins with the galectin-like domain (PF00337) were actively expressed in all analyzed tissues (Table 2). In plants, this domain is present in several galactosyltransferases that form a small gene family (*GALT1-6* in *A. thaliana*) within family 31 of glycosyltransferases (GT) according to CAZy (^20^Henrissat and Davies, 1997). GALT1 (AT1G26810) is a β-1,3-galactosyltransferase that adds galactose to a terminal β-*N*-acetylglucosamine during the formation of Lewis structures in *N*-glycans (Strasser et al., 2007; Showalter and Basu, 2016). The group of GALT1 homologs in flax was extended to four genes (Figure 5, Supplementary File 3). Out of 11 members of the galectin-like family in flax, all were predicted to possess transmembrane domains (Figure 5 and Table 1). Based on the LocTree3 (Table 2) prediction, flax galactosyltransferases with PF00337 domains are localized to membranes of the Golgi apparatus.

Some proteins with lectin domains from several families were predicted to be secreted into the cell wall (Table 2). Several β-galactosidases of the galactose-binding lectin family (PF02140) (Lus10000701, Lus10041798, and Lus10028348) were among them (Table 2). The *in silico* characterization of flax enzymes from this family was performed earlier (Hobson and Deyholos, 2013); lectin domains were found in 22 of the 43 flax β-galactosidases and were always appended to the protein C-terminus.

The only member of the EUL family (PF14200) in the flax genome, *Lus10006551*, was expressed in all analyzed tissues (Table 2). ArathEULS3 protein has been shown to interact with specific *N*-glycans using glycan microarrays representing major glycan structures of glycoproteins and glycolipids (Van Hove et al., 2011).

### Differential Expression of Lectin Genes in Various Stem Tissues

The results from the hierarchical clustering and heatmap analyses of normalized expression values for 302 lectin-encoding genes indicated three groups of samples differing based on the type of cell wall: PCW, SCW, and TCW (Figure 2A). In the current study, the threshold of fold changes ≥ 2 in pairwise comparisons with other sample types (*p* ≤ 0.01, TGR ≥ 16 in at least one sample) was used to consider a gene as differentially expressed.

#### Genes for Proteins With Lectin Domains Upregulated in Tissues With Primary Cell Walls

Several lectin genes were predominantly expressed in SAM, together with other tissues with primary cell walls – cPAR and iFIB (Table 3). Among these tissues, genes for four flax malectins, homologs of *A. thaliana* genes for Di-glucose binding protein with kinesin motor domains were detected: *Lus10025708* and *Lus10035954*, both homologous to *AT2G22610*; *Lus10013714* and *Lus10005582*, both homologous to *AT1G72250*. The latter grouped a separate clade in the malectin family (Figure 3). Both *A. thaliana* homologs, named *MDKIN1* (*At1g72250*) and *MDKIN2* (*At2g22610*) (MALECTIN DOMAIN KINESIN) are expressed in cell division zones and in vasculature; their experimentally established intracellular localization is predominantly associated with nuclei (Galindo-Trigo et al., 2020).

**TABLE 3:** *Linum usitatissimum* genes encoding proteins with lectin domains upregulated in flax stem tissue samples with the primary cell wall.

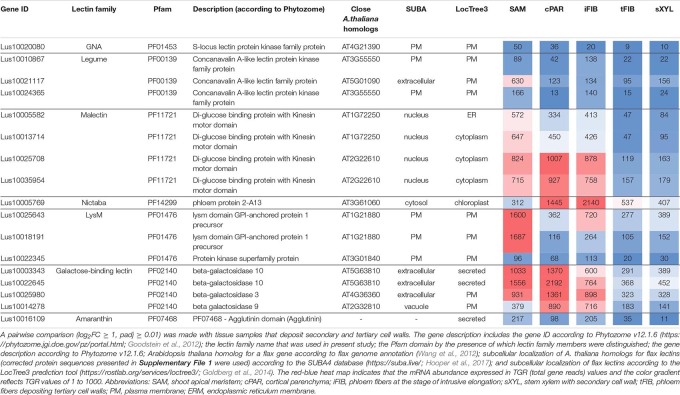

*A pairwise comparison (log_2_FC ≥ 1, padj ≥ 0.01) was made with tissue samples that deposit secondary and tertiary cell walls. The gene description includes the gene ID according to Phytozome v12.1.6 (https://phytozome.jgi.doe.gov/pz/portal.html; Goodstein et al., 2012); the lectin family name that was used in present study; the Pfam domain by the presence of which lectin family members were distinguished; the gene description according to Phytozome v12.1.6; *Arabidopsis thaliana* homolog for a flax gene according to flax genome annotation (Wang et al., 2012); subcellular localization of *A. thaliana* homologs for flax lectins (corrected protein sequences presented in Supplementary File 1 were used) according to the SUBA4 database (https://suba.live/; Hooper et al., 2017); and subcellular localization of flax lectins according to the LocTree3 prediction tool (https://rostlab.org/services/loctree3/; Goldberg et al., 2014). The red-blue heat map indicates that the mRNA abundance expressed in TGR (total gene reads) values and the color gradient reflects TGR values of 1 to 1000. Abbreviations: SAM, shoot apical meristem; cPAR, cortical parenchyma; iFIB, phloem fibers at the stage of intrusive elongation; sXYL, stem xylem with secondary cell wall; tFIB, phloem fibers depositing tertiary cell walls; PM, plasma membrane; ERM, endoplasmic reticulum membrane.*

Three lectin genes were detected with the established thresholds as upregulated predominantly in SAM (Table 3): *Lus10021117* (homolog of *AT5G01090* encoding legume lectin family protein with PF00139 domain), *Lus10018191* and *Lus10025643*, both homologous to AT1G21880 with LysM domain (PF01476). The latter encodes a GPI-anchored protein named LYM1 that belongs to LecP and was characterized as participating in the recognition of bacterial peptidoglycan (Willmann et al., 2011).

Most of the lectins with upregulated gene expression in the samples with primary cell walls were localized in the plasma membrane according to the LocTree3 prediction. The examples included Lus10010867 and Lus10024365, which are homologous to AT3G55550, encoding legume-type lectin receptor kinase LECRKS4 (Bouwmeester and Govers, 2009) (Table 3). Some flax proteins with lectin domains with upregulated expression in cells depositing primary cell walls were predicted to be secreted; these included amaranthin (Lus10016109) and two β-galactosidases (Lus10003343 and Lus10022645, both homologous to AT5G63810) (Table 3). Nineteen flax genes for amaranthin-like lectins and their variable expression in flax tissues were previously characterized (Faruque et al., 2015), *Lus10016109* was designated as *LuALL7*.

#### Genes for Proteins With Lectin Domains Upregulated in Samples Depositing Secondary Cell Walls

Numerous lectins were upregulated in sXYL samples (Table 4) that contain several cell types, all depositing secondary cell wall: vessels, xylem fibers, and xylem parenchyma. sXYL samples were collected in two locations of the stem (Figure 1); the expression data for sXYLa and sXYLb samples were combined and averaged to reveal genes upregulated during secondary cell wall deposition.

**TABLE 4:** *Linum usitatissimum* genes encoding proteins with lectin domains upregulated in flax stem tissue samples with the secondary cell wall.

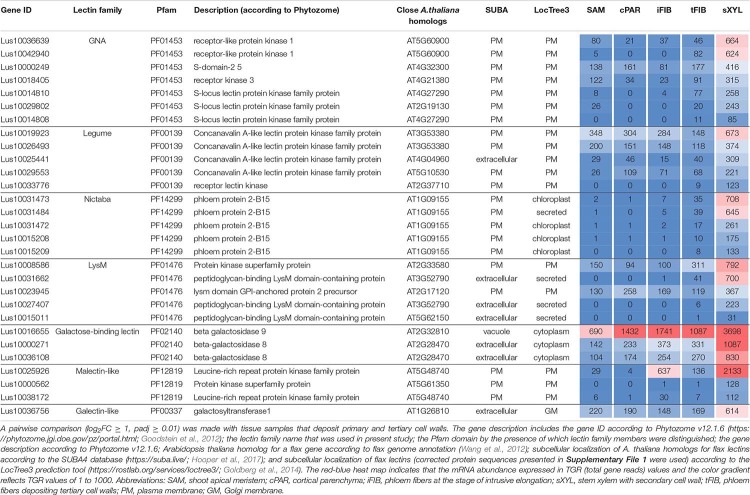

*A pairwise comparison (log_2_FC ≥ 1, padj ≥ 0.01) was made with tissue samples that deposit primary and tertiary cell walls. The gene description includes the gene ID according to Phytozome v12.1.6 (https://phytozome.jgi.doe.gov/pz/portal.html; Goodstein et al., 2012); the lectin family name that was used in present study; the Pfam domain by the presence of which lectin family members were distinguished; the gene description according to Phytozome v12.1.6; *Arabidopsis thaliana* homolog for a flax gene according to flax genome annotation (Wang et al., 2012); subcellular localization of *A. thaliana* homologs for flax lectins according to the SUBA4 database (https://suba.live/; Hooper et al., 2017); and subcellular localization of flax lectins (corrected protein sequences presented in Supplementary File 1 were used) according to the LocTree3 prediction tool (https://rostlab.org/services/loctree3/; Goldberg et al., 2014). The red-blue heat map indicates that the mRNA abundance expressed in TGR (total gene reads) values and the color gradient reflects TGR values of 1 to 1000. Abbreviations: SAM, shoot apical meristem; cPAR, cortical parenchyma; iFIB, phloem fibers at the stage of intrusive elongation; sXYL, stem xylem with secondary cell wall; tFIB, phloem fibers depositing tertiary cell walls; PM, plasma membrane; GM, Golgi membrane.*

**FIGURE 6:**
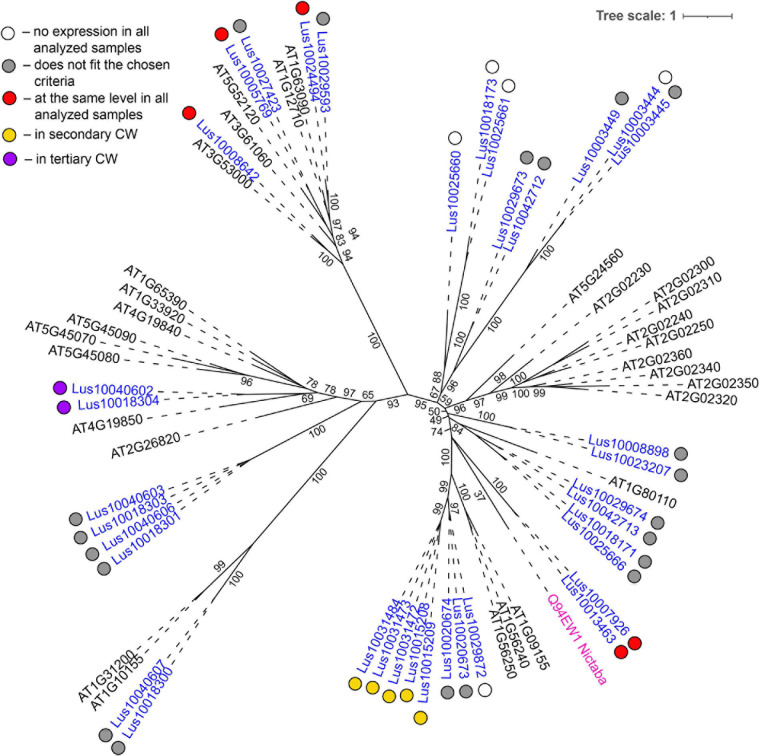
Phylogenetic dendrogram of Nictaba (PF14299) family members of *Arabidopsis thaliana* and *Linum usitatissimum*. The *A. thaliana* gene names are given in black font. The *L. usitatissimum* gene names are given in dark blue font. Originally characterized Nictaba lectin from *Nicotiana tabacum* (Chen et al., 2002) is given in pink font (protein name is given according to the Uniprot database (https://www.uniprot.org/; The UniProt Consortium, 2019). The different colored dots next to *L. usitatissimum* gene names indicate genes with different expression patterns. The criteria used for different expression pattern identification as follow: red, the mean values of tau-score < 0.15 across all samples; yellow, DEGs upregulated in samples with secondary CW; purple dots, DEGs upregulated in samples with tertiary CW. Flax genes with red, yellow, and purple dots are listed in Tables 2, 4, 5, respectively. White dots indicate genes that are not expressed in the analyzed flax samples, and gray dots indicate genes where the expression does not fit the chosen criteria (tau-score < 0.15 or DEGs) for a certain expression pattern (expression values for these genes are given in Supplementary File 3). Numbers indicate the ultrafast bootstrap support values for some branches. Abbreviations: DEGs, differentially expressed genes, a pairwise comparison log_2_FC ≥ 1, padj ≥ 0.01, CW, cell wall.

The most common groups identified were lectins from GNA, legume, LysM, malectin, and Nictaba (PP2) families. Of these, only the latter family was not predicted to be localized to the plasma membrane or cell wall. Five genes with a Nictaba domain were upregulated in sXYL samples (*Lus10031484*, *Lus10031473*, *Lus10031472*, *Lus10015209*, and *Lus10015208*) and belong to a separate clade in the phylogenetic tree (Figure 6, yellow dots). All of these five genes were recognized as the homologs of *AT1G09155*, which encodes the F-box containing phloem protein PP2-B15 (Table 4).

Members of GNA, legume, LysM-type, and malectin-like lectin families that were predominantly expressed in xylem were all predicted to be localized to the plasma membrane or secreted (Table 4). Among the GNA lectins detected as upregulated in sXYL samples, the most pronounced expression was observed for *Lus10036639* and *Lus10042940*, which are both homologous to *AT5G60900*, encoding RLK1a, a receptor like kinase with leucine-rich repeats (Table 4). Six genes of legume lectin receptor kinases were activated in xylem tissues [flax homologs of *LECRK-VIII.1* (*AT3G53380*), *LECRK-VII.1* (*AT4G04960*), *LECRK-IX.1* (*AT5G10530*), and *LECRK-IV.1* (*AT2G37710*)] (Table 4).

**FIGURE 7:**
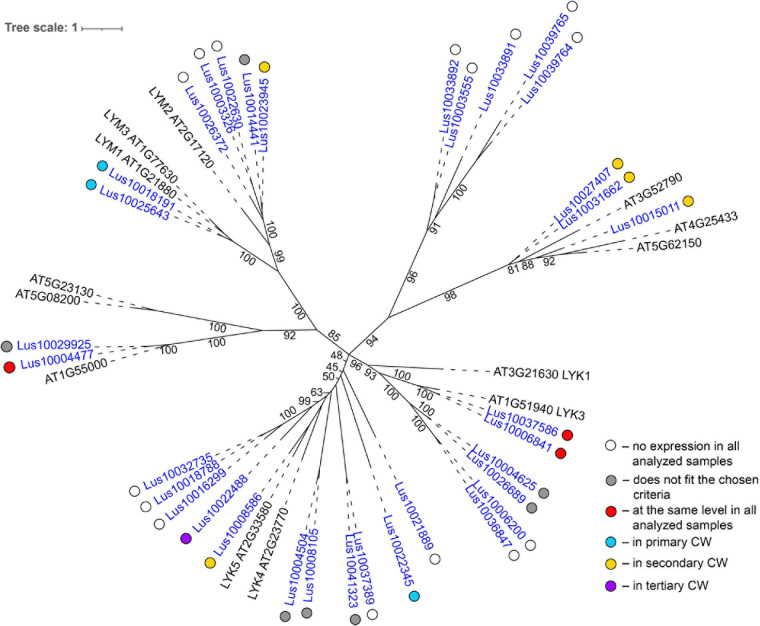
Phylogenetic dendrogram of LysM (PF01453) lectin family members of *Arabidopsis thaliana* and *Linum usitatissimum*. The *A. thaliana* gene names are given in black font; additional gene names are given according to Shinya et al. (2012) and Wan et al. (2012). The *L. usitatissimum* gene names are given in dark blue font. The different colored dots next to *L. usitatissimum* gene names indicate genes with different expression patterns. The criteria used for different expression pattern identification as follow: red, the mean values of tau-score < 0.15 across all samples; light blue, DEGs upregulated in samples with primary CW; yellow, DEGs upregulated in samples with secondary CW; purple dots, DEGs upregulated in samples with tertiary CW. Flax genes with red, light blue, yellow, and purple dots are listed in Tables 2–5, respectively. White dots indicate genes that are not expressed in the analyzed flax samples, and gray dots indicate genes where the expression does not fit the chosen criteria for a certain expression pattern (expression values for these genes are given in Supplementary File 3). Numbers indicate the ultrafast bootstrap support values for some branches. Abbreviations: DEGs, differentially expressed genes, a pairwise comparison log_2_FC ≥ 1; padj ≥ 0.01, CW, cell wall.

Several genes for lectins with a LysM motif that localized to the plasma membrane were upregulated in sXYL samples (Table 4). These included Lus10008586, which is homologous to *A. thaliana* LYK5 (AT2G33580) – a protein with LecRLK type architecture, and Lus10023945, which is homologous to LYM2 (AT2G17120), a GPI-anchored LecP type protein. LYK5 and LYM2 belong to distant clades (Figure 7). Three LecRLP type lectins predicted to be secreted into the cell wall showed higher gene expression levels, namely *Lus10031662*, *Lus10027407* (Table 4), and *Lus10015011* (Supplementary File 3). These three LecRLP type lectins clustered together on the phylogenetic tree (Figure 7). The functions of their closest *A. thaliana* homologs, AT3G52790, AT4G25433 and AT5G62150 are unknown.

Three genes for protein kinases with a malectin-like domain (PF12819), *Lus10000562* (homologous to *AT5G61350*), *Lus10025926*, and *Lus10038172* (both homologous to *AT5G48740*), showed an increased mRNA abundance in sXYL tissues compared to other samples (Table 4). Galactosyltransferase (Lus10036756) with a galectin-like domain that is homologous to GALT1 from *A. thaliana* also showed pronounced expression. GALT1 is a β-d-1,3-galactosyltransferase that is involved in the formation of *N*-glycans (Strasser et al., 2007). Expression of the specific isoform indicates some peculiarities of *N*-glycans in xylem tissue; however, nothing is currently known about *N*-glycoproteins that are specific to secondary cell walls.

#### Genes for Proteins With Lectin Domains Upregulated in Samples Depositing Tertiary Cell Wall

Tissue- and stage-specific expression of lectin genes was exemplified by flax phloem fibers isolated from the stem at an advanced stage of specialization when they deposited tertiary cell wall. Fibers were isolated at two locations in the stem, both located below the snap point (Figure 1; Gorshkova et al., 2003); the expression data for tFIBa and tFIBb samples were combined and averaged to reveal genes upregulated at the stage of tertiary cell wall deposition. Based on the expression profile, lectins from several families were considerably upregulated in fibers at this stage of development (Table 5).

**TABLE 5:** *Linum usitatissimum* genes encoding proteins with lectin domains upregulated in flax stem tissue samples with the tertiary cell wall.

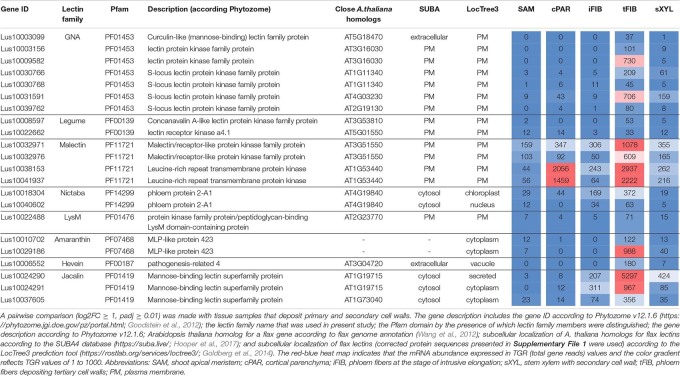

*A pairwise comparison (log2FC ≥ 1, padj ≥ 0.01) was made with tissue samples that deposit primary and secondary cell walls. The gene description includes the gene ID according to Phytozome v12.1.6 (https://phytozome.jgi.doe.gov/pz/portal.html; Goodstein et al., 2012); the lectin family name that was used in present study; the Pfam domain by the presence of which lectin family members were distinguished; the gene description according to Phytozome v12.1.6; Arabidopsis thaliana homolog for a flax gene according to flax genome annotation (Wang et al., 2012); subcellular localization of *A. thaliana* homologs for flax lectins according to the SUBA4 database (https://suba.live/; Hooper et al., 2017); and subcellular localization of flax lectins (corrected protein sequences presented in Supplementary File 1 were used) according to the LocTree3 prediction tool (https://rostlab.org/services/loctree3/; Goldberg et al., 2014). The red-blue heat map indicates that the mRNA abundance expressed in TGR (total gene reads) values and the color gradient reflects TGR values of 1 to 1000. Abbreviations: SAM, shoot apical meristem; cPAR, cortical parenchyma; iFIB, phloem fibers at the stage of intrusive elongation; sXYL, stem xylem with secondary cell wall; tFIB, phloem fibers depositing tertiary cell walls; PM, plasma membrane.*

Three genes encoding proteins with jacalin-like domains (PF01419), *Lus10024290*, *Lus10024291* (both homologs of *AT1G19715*), and *Lus10037605* (homolog of *AT1G73040*) were notably upregulated in tFIB. The flax genome contains only four jacalin genes, three of which were detected as highly activated in fibers depositing tertiary cell walls. No sequences for signal peptides and transmembrane domains were detected in flax jacalins (Table 1), however, the jacalin encoded by *Lus10024290* with the highest expression level was predicted to be secreted into the cell wall by LocTree3 program (Table 5). The extracellular jacalin Horcolin devoid of a signal peptide was biochemically isolated from barley coleoptiles (Grunwald et al., 2007).

**FIGURE 8:**
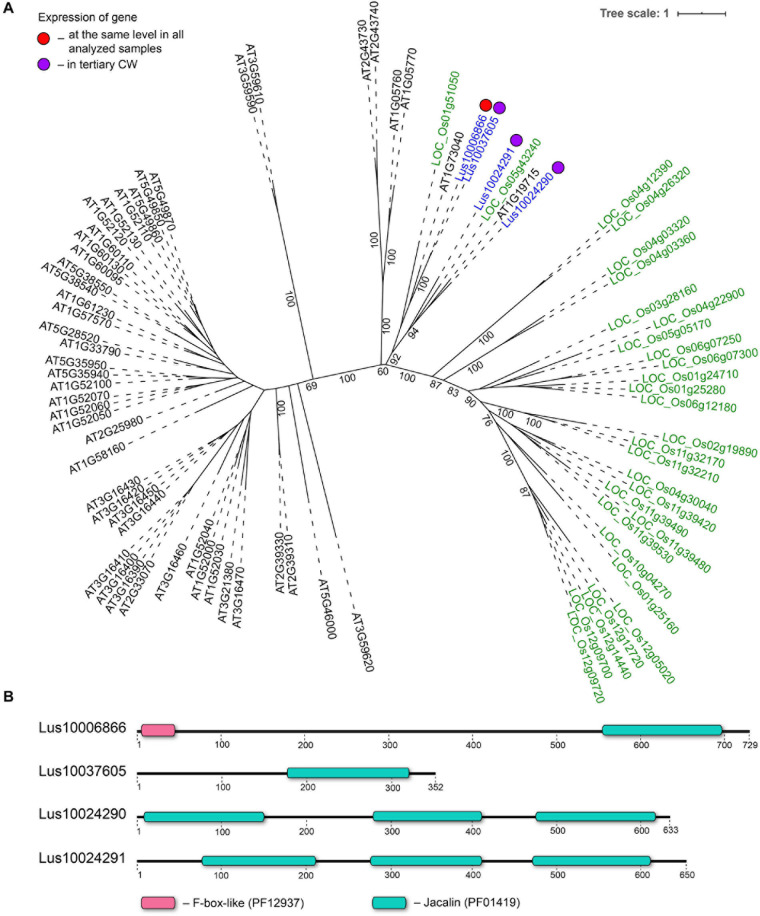
Phylogenetic dendrogram **(A)** of jacalin (PF01419) lectin family members of *Arabidopsis thaliana*, *Linum usitatissimum*, and *Oryza sativa* (Japonica Group) and domain size and distribution **(B)** in their protein sequences. **(A)** The *A. thaliana* gene names are given in black font, *L. usitatissimum* gene names are given in dark blue font, and *O. sativa* gene names are given in green font. The different colored dots next to *L. usitatissimum* gene names indicate genes with different expression patterns. The criteria used for different expression pattern identification as follow: red, the mean values of tau-score < 0.15 across all samples; purple dots, DEGs upregulated in samples with tertiary CW. Flax genes with red and purple dots are listed in Tables 2, 5, respectively. Numbers indicate the ultrafast bootstrap support values for some branches. **(B)** The domain size and distribution are shown according to results of domain search using the InterProScan tool of the InterPro database (https://www.ebi.ac.uk/interpro/; Mitchell et al., 2019). Numbers indicate the length of amino acid sequences. Abbreviations: DEGs, differentially expressed genes, a pairwise comparison log_2_FC ≥ 1; padj ≥ 0.01, CW, cell wall.

On the dendrogram, all flax jacalins localized within a small cluster that combined several sequences of rice and *A. thaliana* jacalins (Figure 8A). The jacalin domain is often present in proteins as tandem repeats, which can be combined with other domains (Eggermont et al., 2017). In accordance, two flax lectins belonging to this protein family had three jacalin domains in a row, while two others had only one (Figure 8B). The jacalin with a stable character of expression (*Lus10006866*, Table 2) had a jacalin domain combined with the F-box, while those upregulated in fibers depositing tertiary cell wall have only jacalin domains (Figure 8B).

The *LuALL4* (*Lus10029186*) and *LuALL11* (*Lus10010708*) members of the amaranthin family (PF07468) (Faruque et al., 2015) were upregulated in the fibers depositing tertiary cell wall. These lectins, which are distinct from *LuALL7* mainly expressed in tissues with primary cell wall, have a pathogenesis-related protein Bet v I domain. The four flax genes belonging to the Nictaba family were also upregulated at the tertiary cell wall formation. Two of them are homologs to the *A. thaliana* phloem protein PP2-A1 (AT4G19840), which contains only a Nictaba domain and is part of the phloem protein bodies in the sieve elements. Recombinant protein production and glycan array analysis demonstrated the binding of PP2-A1 to *N*-acetylglucosamine oligomers, high-mannose *N*-glycans, and 9-acyl-*N*-acetylneuraminic sialic acid (Beneteau et al., 2010).

Over a half of lectins upregulated in fibers with tertiary cell walls were predicted to localize at the plasma membrane (Table 5). Among them, the most numerous were LecRLKs with a GNA domain (PF01453) and genes belonging to the malectin (PF11721) family. *Lus10009582* encoding the lectin kinase with the highest expression among GNA-type lectins, was specifically expressed in tFIB samples (Table 5). Two genes from the malectin family, *Lus10038153* and *Lus10041937*, which are both homologous to *AT1G53440* that encodes leucine-rich repeat transmembrane protein kinases, were additionally highly expressed in cortical parenchyma. Two other malectins, Lus10032971 and Lus10032976 are homologous to FERONIA (AT3G51550), which is the receptor-like kinase in *A. thaliana* demonstrated to interact with cell wall pectins and to be involved in numerous developmental processes (Li et al., 2018).

### Changes in Lectin Gene Expression Upon Plant Gravistimulation

To characterize the expression of lectin genes during induced gravitropic response, we used a dataset including samples from the control non-inclined plants (sXYLb and tFIBb) and samples from the region of the formation of stem curvature developed to return the inclined plants to vertical position (Ibragimova et al., 2017; Gorshkov et al., 2018). Each stem segment was separated into pulling (PUL) and opposite (OPP) sides both in the xylem and in the phloem (Figure 1); the outer peel of each stem side was used to isolate phloem fibers.

**FIGURE 9:**
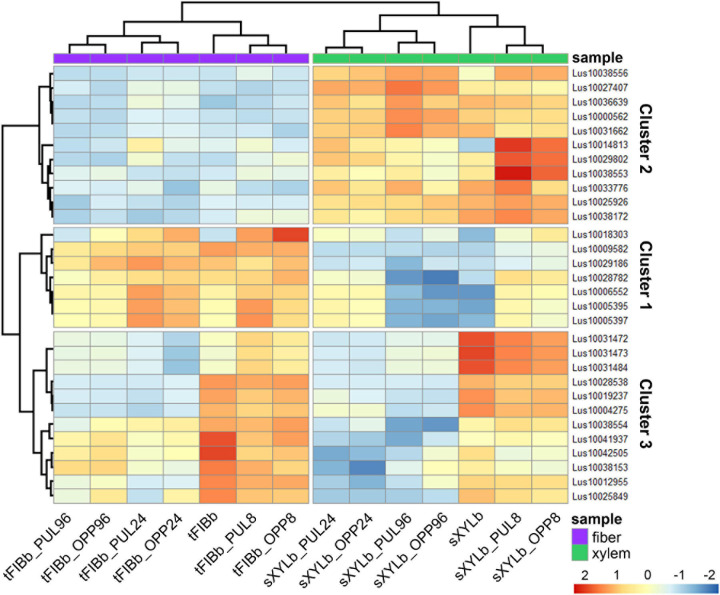
Changes in expression of *Linum usitatissimum* genes for proteins with lectin domains during graviresponse. A heatmap of the top 30 lectin genes with the highest variance across samples from control plants (sXYLb and tFIBb) and from the segment with gravibending stems at various time points. Sample information is shown with colored bars at the top of the heatmap and abbreviations at the bottom. The color of the heatmap corresponds to the amount by which the variation of gene expression deviates from the gene’s mean variance across all samples. tFIBb and sXYLb – isolated fibers with TCW and stem xylem with SCW of non-inclined plant, respectively; tFIBb_PUL8, tFIBb_PUL24, tFIBb_PUL96 – isolated fibers with TCW from pulling side of flax stem after 8, 24, 96 h of gravibending, respectively; tFIBb_OPP8, tFIBb_OPP24, tFIBb_OPP96 – isolated fibers with TCW from opposite side of flax stem after 8, 24, 96 h of gravibending, respectively; sXYLb_PUL8, sXYlb_PUL24, sXYLb_PUL96 – pulling side of stem xylem with SCW after 8, 24, 96 h of gravibending, respectively; sXYLb_OPP8, sXYlb_OPP24, sXYLb_OPP96– opposite side of stem xylem with SCW after 8, 24, 96 h of gravibending. Abbreviations: SCW, secondary cell wall; and TCW, tertiary cell wall.

To identify the most prominent representatives of lectins expressed during gravitropic response, we performed gene clustering based not on the absolute expression strength but rather on the amount by which each gene deviates in a specific sample from the gene’s average across all samples. Hence, we centered the values of each gene in the samples and built a heat map. The top 30 genes with the highest variance of expression levels across samples are shown in the Figure 9 and Table 6.

**TABLE 6:** Changes in expression of *Linum usitatissimum* genes for proteins with lectin domains during graviresponse.

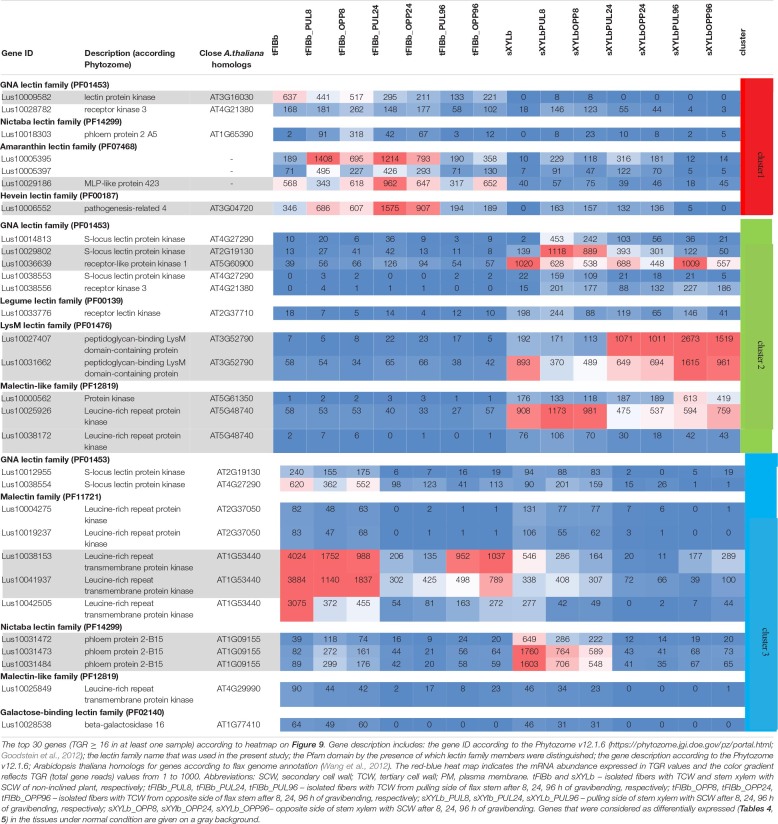

*The top 30 genes (TGR ≥ 16 in at least one sample) according to heatmap on Figure 9. Gene description includes: the gene ID according to the Phytozome v12.1.6 (https://phytozome.jgi.doe.gov/pz/portal.html; Goodstein et al., 2012); the lectin family name that was used in the present study; the Pfam domain by the presence of which lectin family members were distinguished; the gene description according to the Phytozome v12.1.6; *Arabidopsis thaliana* homologs for genes according to flax genome annotation (Wang et al., 2012). The red-blue heat map indicates the mRNA abundance expressed in TGR values and the color gradient reflects TGR (total gene reads) values from 1 to 1000. Abbreviations: SCW, secondary cell wall; TCW, tertiary cell wall; PM, plasma membrane. tFIBb and sXYLb – isolated fibers with TCW and stem xylem with SCW of non-inclined plant, respectively; tFIBb_PUL8, tFIBb_PUL24, tFIBb_PUL96 – isolated fibers with TCW from pulling side of flax stem after 8, 24, 96 h of gravibending, respectively; tFIBb_OPP8, tFIBb_OPP24, tFIBb_OPP96 – isolated fibers with TCW from opposite side of flax stem after 8, 24, 96 h of gravibending, respectively; sXYLb_PUL8, sXYlb_PUL24, sXYLb_PUL96 – pulling side of stem xylem with SCW after 8, 24, 96 h of gravibending, respectively; sXYLb_OPP8, sXYlb_OPP24, sXYLb_OPP96– opposite side of stem xylem with SCW after 8, 24, 96 h of gravibending. Genes that were considered as differentially expressed (Tables 4, 5) in the tissues under normal condition are given on a gray background.*

Results of the clustering analysis showed three expression patterns of lectin genes in plants subjected to gravistimulation (Figure 9 and Table 6). Cluster 1 was dominated by transcripts abundant in phloem fibers (Figure 9 and Table 6, Cluster 1), moreover, 3 out 7 genes (genes for GNA lectin *Lus10009582*, for amarantin *Lus10029186*, and for hevein *Lus10006552*) of this cluster were already mentioned as genes specifically expressed in tFIB samples (see section “Genes for Proteins With Lectin Domains Upregulated in Samples Depositing Tertiary Cell Wall,” Table 5). Cluster 2 was represented by lectin genes, which were up-regulated in xylem (Figure 9 and Table 6, Cluster 2), and most of genes in this cluster were designated as DEGs upregulated in sXYL samples fibers (see section “Genes for Proteins With Lectin Domains Upregulated in Samples Depositing Secondary Cell Walls”). Cluster 3 grouped transcripts that had high expression in analyzed tissues of both non-inclined plants and gravistimulated plants (8 h after inclination) and further were drastically decreased (Figure 9 and Table 6, Cluster 3); this cluster included 3 genes for lectins of Nictaba family (*Lus10031472*, *Lus10031473*, *Lus10031484*) that were upregulated in sXYL samples (see section “Genes for Proteins With Lectin Domains Upregulated in Samples Depositing Secondary Cell Walls”) and 2 genes for lectins of Malectin family (*Lus10038153* and *Lus10041937*) that were upregulated in tFIB samples (see section “Genes for Proteins With Lectin Domains Upregulated in Samples Depositing Tertiary Cell Wall,” Table 5).

The expression levels of genes encoding proteins with lectin domains from different families changed substantially after gravistimulation (Figure 9 and Table 6). The expression patterns of lectin genes that differed between various tissues (tFIB and sXYL) and between various time points in the course of gravitropic response development (8 h, 24 h, and 96 h) were revealed.

## Discussion

### The Set of Proteins Within Some Lectin Families Varies Between Plant Species

The total number of lectin genes in the flax genome, over 400 genes, is among the highest described for a plant species (Van Holle et al., 2017). The proportion of the number of genes in different lectin families is similar to that described in the literature for other higher plant species (Jiang et al., 2010; Bellande et al., 2017; Van Holle et al., 2017). The most numerous lectin families are GNA-type (112 representatives) and legume-type lectins (72 representatives) that are characteristic for plants and were historically the earliest described (Table 1). Some lectin families have quite variable numbers of members between plant species. An extreme example is the amaranthin-like lectin family, which is not ubiquitous among angiosperms. Many taxonomically distant plant species (e.g., *A. thaliana*, soybean, poplar, and rice) do not have amaranthin-like lectins in their genomes, while others (apple, hemp, maize, cucumber, etc.) have amaranthin-like lectins that are represented by multigene families that account for up to 24 members (Dang et al., 2017); flax has one of the largest families (Faruque et al., 2015; Table 1). On the other hand, the group of flax jacalin-like lectins consists of only four genes, in contrast to the several dozen representatives in the genomes of rice and *A. thaliana*. In *A. thaliana*, the jacalin family is among the largest, comprising almost a quarter of all lectins (Van Holle et al., 2017).

In the phylogenetic trees of lectin families, large groups of members from different plant species may be fully separated. The large clade of 35 malectin-like proteins in *A. thaliana*, which includes MEE39 and PAM74, does not have a flax counterpart (Figure 3). Similarly, flax is lacking some large clades of legume-type lectins (Figure 4). The distribution of jacalins present in *A. thaliana* and rice genomes on the phylogenetic tree is almost mutually exclusive (Van Holle et al., 2017, Figures 8A,B); flax jacalins are localized only within a small group that is mutual for all three species. The divergent sets of proteins within several lectin families indicate that these proteins are among those determining the specificity of development and reaction to biotic and abiotic stressors between plant species.

### Proteins With Lectin Domains Are Widely Involved in the Life of Plants

The expression of proteins with lectin domains in different tissues of the same organ was analyzed using flax stem. Three quarters of proteins with lectin domains present in the flax genome were expressed in stem tissues (TGR > 50), indicating the wide involvement of lectins in the everyday life of plant organisms (Table 1). In several lectin families that had a small number of members, such as calreticulins and proteins with C-type and EUL domains, all members were expressed in all analyzed tissues, suggesting the involvement in basic processes. Calreticulins, for example, are located in endoplasmic reticulum and ensure proper folding and quality control of the synthesized secretory and membrane glycoproteins before exiting the endoplasmic reticulum (Lannoo and Van Damme, 2014). Unlike classical chaperones, which interact with the peptide part of the substrates, CANX and CALR bind to oligosaccharide chains of glycoproteins. A high proportion of the expressed genes but with variable expression levels in different samples was revealed for most of the other lectin families (Supplementary File 3), including proteins with GNA-type, legume-type, LysM, malectin, Nictaba, galactose-binding lectin, and galectin-like domains.

The function of lectins is often associated with a plant’s reaction to pathogens (De Hoff et al., 2009; Vandenborre et al., 2011). However, plant immunity and development often rely on similar or overlapping cellular mechanisms for signal recognition and transduction (Jamieson et al., 2018). A genome-wide analysis of all flax proteins with lectin domains demonstrates that lectins are actively and differentially expressed both under normal growth conditions and under abiotic stress conditions including during gravitropic response. The expression of flax lectins is specific for various tissues and also for the different stages of cell development such as in phloem fibers at intrusive elongation and tertiary cell wall deposition stages (Supplementary File 3). Though the flax plants sampled in this study were grown under non-sterile conditions and it cannot be excluded that the expression of some lectins was associated with the reaction to microorganisms, the high proportion of transcribed genes for lectins, their differential expression in various tissues and at various stages of the distinct cell type development, together with the dynamic changes of expression under the influence of abiotic factors suggest that proteins with lectin domains are widely involved in plant development, tissue specialization, and reaction to the effect of abiotic stressors.

Proteins with the same lectin domain can be localized within different subcellular structures and fulfill different functions. Most of the lectins are chimerolectins, i.e., proteins with additional domains (Bellande et al., 2017; Eggermont et al., 2017). The combinations of various domains in a protein are variable, especially considering proteins from organisms in different kingdoms. For example, there are vast differences in the proteins with galectin-like domains (PF00337). In animals, the galectins are small, mainly extracellular proteins secreted by a non-classical exocytic pathway; they contribute to cell–cell and cell–matrix interactions due to the processes of multivalent carbohydrate recognition combined with galectin di- and oligomerization (Dodd and Drickamer, 2001; Nabi et al., 2015). In plants, the same characteristic domain is present within several galactosyltransferases, like in *A. thaliana* and flax (Figure 5). According to the current definitions, if taken strictly, the same domain should be considered lectin in animals and CBM in plants. Moreover, differences can be detected even within a family. In *A. thaliana*, the protein AT4G19810 from the class V chitinase-related agglutinin (CRA) family possesses chitinase activity and cannot be referred to as a lectin by definition (Eggermont et al., 2017). Lectins are primarily searched for based on corresponding protein domains, especially in full genomic studies (Bellande et al., 2017; Eggermont et al., 2017, current study); however, this method cannot guarantee the absence of enzymatic activity in protein. This complicates the identification and definition of plant lectins, and the lists of plant lectin families in classifications suggested by various authors do not fully match (Table 1). In our study, we have considered all proteins with lectin domains, since the presence of a carbohydrate-recognizing domain in a protein may add some important features to its function.

### Numerous Lectins Have the Potential to Interact With Cell Wall Glycans

Many plant lectins function in the cytoplasm and nucleus, whereas others are exposed to the cell surface because they are secreted into the cell wall or anchored to the plasma membrane and have an extracellular part of the protein. The interaction of some lectins with the cell wall or with certain cell wall glycans was demonstrated by various approaches. The direct association with polygalacturonic acid could be identified for plasma membrane receptor-like kinase with the malectin domain (Feng et al., 2018). The association with cell wall components of another transmembrane protein that has a malectin domain but no kinase domain was demonstrated at the analysis of plasmolyzed cells in lateral roots of *A. thaliana* (Schürholz, 2019). Similar experiments showed that GFP-tagged THE1 and FER are also tightly bound to the cell wall (Hématy et al., 2007; Li et al., 2018). The lectin receptor kinase with a legume-type domain is involved in maintaining cell wall-plasma membrane adhesion through protein–protein interactions with the participation of the tripeptide motifs Arg-Gly-Asp (RGD) (Gouget et al., 2006). Lectins of various architectural types, such as LecP, LecRLPs, and LecRLKs, are readily found by proteomic approaches both in the cell wall and plasma membrane (Jamet et al., 2008; Bellande et al., 2017). The secretion of some lectins without signal peptides into the cell wall via non-classical way was demonstrated for animal galectins (Delacour et al., 2009) and plant proteins with EUL (Jamet et al., 2008; Dubiel et al., 2020) and jacalin-like (Pinedo et al., 2012, 2015) domains.

The hierarchical clustering grouped all analyzed samples according to the cell wall type (Figure 2A). Phloem fibers at the intrusive elongation stage were grouped together with other tissues depositing primary cell wall rather than together with phloem fibers at the later stage of development when they deposit tertiary cell wall. The majority of genes upregulated in tissues with distinct cell wall types encode lectins localized at the plasma membrane or secreted into the apoplast as predicted by LocTree3 service (Tables 3–5). Thus, the encoded proteins have the potential to directly interact with cell wall glycans.

A characteristic feature of lectins is di- or tetramerization that may involve homo- and hetero-interactions (Müller et al., 2016). Lectins of various architectural types can take part in such interactions. For example, RLPs were suggested to function as a specificity switch for ligand-receptor recognition (Jamieson et al., 2018). In addition, interactions of proteins with lectin domains depend on ligand binding. Glycan sequences characteristic to certain cell wall types and lectins that are specifically upregulated and/or are present in tissues with various cell wall types may provide the platform on which to arrange specific signaling complexes. This is indicated by the comprehensive analysis of all proteins with lectin domains that reveals the specific expression patterns coupled to cell wall type in various flax stem tissues (Tables 3–5).

### Differentially Expressed Flax Lectins Between Samples With Distinct Cell Wall Types

Proteins with lectin domains that are differentially expressed in tissues with distinct cell wall types belong to various lectin families (Tables 3–5). GNA-type lectins comprise the largest lectin family in flax (Table 1), same as in many other plant species (Van Holle et al., 2017). GNA-type lectins have a carbohydrate-binding domain of approximately 150 amino acids, which binds D-mannose and has a conserved mannose-binding motif Q-X-D-X-N-X-V-X-Y (Van Damme et al., 2008; Shimokawa et al., 2012). Genes for GNA-type lectins were actively expressed in flax stem tissues (Table 1) but were rather poorly related to cell wall type, since their numbers among the differentially expressed tissues were low (Tables 3–5). Most of the GNA-type lectins have expression patterns that do not overcome the thresholds used in the current paper and are marked by gray dots in Supplementary File 4. No representative of this family was upregulated in tissues depositing primary cell wall. However, several lectins were detected for the tertiary cell wall. *Lus10009582* and *Lus10031591*, which encode the LecRLKs with the highest expression in fibers among GNA-type lectins, were quite specifically expressed in tFIB samples (Table 5). Several lectins with the LysM motif were upregulated in sXYL samples in the secondary cell wall deposition stage (Table 4). These lectins include proteins with the LecRLK type of architecture that were localized to the plasma membrane, namely, Lus10023945 (homologous to AT2G17120 (LYM2) and Lus10008586 (AT2G33580, LYK5), which belong to distant clades, and three LecRLP type lectins predicted to be secreted into the cell wall, Lus10031662, Lus10027407, and Lus10015011, which all cluster together in the phylogenetic tree (Figure 7).

The legume-type lectin family had representatives upregulated in tissues with each cell wall type. LecRK from this family was differentially expressed in samples with primary, secondary, and tertiary cell walls. For example, Lus10010867 and Lus10024365, which are homologous to AT3G555530, encoding the legume-type lectin receptor kinase LECRKS7 (Bouwmeester and Govers, 2009), were upregulated in tissues with primary cell walls (Table 3). The set of genes for legume-type lectin receptor kinases were activated in sXYL samples with secondary cell wall (Table 4). All of these kinases were predicted to be localized at the plasma membrane. However, information on the different properties of distinct family representatives is scarce, which is also true for many proteins with lectin domains from other families. This limits the discussion of the functional relevance of the changes in gene expression among lectins.

The most differential expression patterns among the proteins with lectin domains in samples with distinct cell wall types were observed for proteins with jacalin-like and malectin (together with malectin-like) domains, as well as for β-galactosidases with lectin domains. Out of four genes for proteins with jacalin-like domain, three were specifically upregulated in fibers depositing tertiary cell wall (Table 5). The fourth gene, distinguished by the presence of F-box (Figure 8B), was expressed in all analyzed tissues (Table 2). The encoded proteins do not have signal peptides and are considered nucleocytoplasmic mannose-binding proteins (Van Holle and Van Damme, 2019); however, research supports cell wall localization and secretion of such proteins via a non-classical pathway (Grunwald et al., 2007; Pinedo et al., 2012, 2015). Flax jacalin encoded by Lus10024290 was predicted to be secreted into the cell wall (Table 5).

Both malectin and malectin-like domains are found in proteins of organisms from various kingdoms; however, plants have many more malectin, malectin-like, legume, and GNA-type domains compared with organisms from other kingdoms (Bellande et al., 2017). In animals, malectin is a well-characterized membrane-anchored endoplasmic reticulum protein that recognizes and binds Glc2-*N*-glycan, thus playing a role in the early stages of protein *N*-glycosylation (Schallus et al., 2008). In plants, this domain is found in a number of receptor kinases localized to the plasma membrane (Franck et al., 2018), indicating that malectin domains have different functions in different kingdoms. The expression patterns of various genes for malectin and malectin-like domains in flax tissues are very diverse (Tables 2–5). Genes for four flax malectins, which are homologs of *A. thaliana* genes for di-glucose binding protein with a kinesin motor domain, grouped as a separate clade in the malectin family (Figure 3). All of them were upregulated in tissues with primary cell wall. Two other malectins, Lus10032971 and Lus10032976 are homologous to FERONIA (AT3G51550) – a receptor-like kinase that was demonstrated to interact with cell wall pectins and to be involved in numerous developmental processes (Li et al., 2016). In *A. thaliana* seedlings, FERONIA was identified as a key regulator in mechano-sensing (Shih et al., 2014; Doblas et al., 2018). In flax, the expression of FERONIA homologs was highly up-regulated in fibers depositing tertiary cell wall. This cell wall type is deposited only in fibers and is considered to have a special mechanical function by providing tension in cellulose microfibrils (Gorshkova et al., 2018a; Almeras et al., 2020). The presence of specific types of pectins is characteristic for tertiary cell walls (Mikshina et al., 2013). Thus, the enhanced expression of FERONIA-like mechano-sensors is very relevant to the composition and function of fibers with tertiary cell wall.

Several β-galactosidases with a lectin domain had expression patterns coupled to the cell wall type; for example, four of them were upregulated in tissues with primary cell wall. All plant β-galactosidases belong to GH35 in CAZy database (Chandrasekar and van der Hoorn, 2016). In flax, among the proteins with the Glyco_hydro_35 (PF01301) domain, the main catalytic domain of GH-35 β-galactosidases, only a portion also had the PF02140 domain (24 out of 43). Similarly, this was observed in jute (only six out of 11 β-galactosidases had galactose-binding lectin domains; Satya et al., 2018), tomato, and *A. thaliana* (12 out of 17 and 10 out of 17, respectively; Chandrasekar and van der Hoorn, 2016), while the lectin domain was present in all 17 β-galactosidases detected in peach (Guo et al., 2018). The β-galactosidases (*Lus10028848* and *Lus10008974*) that are known as key players in the tertiary cell wall structure of flax fibers (Roach et al., 2011; Gorshkova et al., 2018a) are devoid of the PF02140 domain. This domain, if present, is a C-terminal domain that is homologous to galactose- and rhamnose-binding animal lectins, which is designated a SUEL (Sea Urchin Egg Lectin)-type carbohydrate-binding domain. The structure and function of this domain in rice β-galactosidase, OsBGal1, was studied in detail (Rimlumduan et al., 2016). Although the binding of galactose and rhamnose was predicted for OsBGal1 based on amino acid sequence homology with SUEL lectin, binding to rhamnose, galactose, glucose, β-1,4-galactobiose, and raffinose was not observed in NMR experiments. Thus, experimental identification of carbohydrate specificity of the PF02140 domain is still required, as is the identification of carbohydrate specificity for the vast majority of plant lectins. Moreover, some lectins, like Nictaba, have many promiscuous carbohydrate binding sites and are capable of interacting with different carbohydrate motifs (Delporte et al., 2015).

Altogether, the expression of many proteins with lectin domains is related to the formation of the carbohydrate-enriched compartment of plant cell – the wall. Moreover, distinct cell wall types have both shared and “personal” cell wall-related proteins with lectin domains, the expression of the latter is highly upregulated at the formation of PCW, SCW, or TCW. This can be coupled to the peculiarities of glycan composition in various cell wall types. Thus, in-depth further studies of lectin specificity in relation to plant cell wall polymers are highly demanded. The distinct combinations of lectins and complex carbohydrates may give rise to specific regulatory modes characteristic for certain cell wall types. The diversity of the specific recognition systems on the cell surface of various tissues may enrich the complex regulation of plant organism development and reaction to abiotic stress.

## Data Availability Statement

The 40 flax transcriptome libraries considered in this study were previously obtained by us and deposited in the Sequence Read Archive (SRA) as BioProjects (PRJNA475325 and PRJNA631357). The four RNA-seq datasets from the stem apex (PRJNA229810, Zhang and Deyholos, 2016) were downloaded from the European Nucleotide Archive (https://www.ebi.ac.uk/ena) as fastq-files of raw data.

## Author Contributions

NP, OP, and TG thought up and designed the study. OG, AN, NP, NM, and TG performed the experiments analyzed the obtained data and jointly discussed them. TG and NP wrote the manuscript. The illustrations and tables were prepared by NP (Figure 1 and Tables 1–6, Supplementary Files 1, 2), OG (Figures 2, 9, Tables 2–6, Supplementary File 3), AN (Figures 4–8, Supplementary Files 1, 4), and NM (Supplementary Files 1, 2). All authors reviewed and approved the manuscripts.

## Conflict of Interest

The authors declare that the research was conducted in the absence of any commercial or financial relationships that could be construed as a potential conflict of interest.

## Footnotes

1 https://www.ebi.ac.uk/ena

2 https://jgi.doe.gov/data-and-tools/bbtools/bb-tools-user-guide/bbduk-guide/

3 http://pfam.xfam.org/

4 https://phytozome.jgi.doe.gov/pz/portal.html

5 https://www.ncbi.nlm.nih.gov/Structure/cdd/wrpsb.cgi

6 https://www.uniprot.org/

7 http://bioinf.uni-greifswald.de/augustus/

8 http://www.softberry.com/berry.phtml?topic=fgenesh&group=programs&subgroup=gfind

9 http://www.cbs.dtu.dk/services/SignalP/

10 http://www.predisi.de/home.html

11 http://ipsort.hgc.jp/index.html

12 http://www.cbs.dtu.dk/services/TMHMM/

13 https://www.ebi.ac.uk/interpro/

14 https://suba.live/

15 https://rostlab.org/services/loctree3/

16 https://rdrr.io/github/roonysgalbi/tispec

17 https://www.ebi.ac.uk/Tools/msa/clustalo/

18 https://itol.embl.de/

19 https://pfam.xfam.org/

20 http://www.cazy.org/

## Supplementary Material

Supplementary File 1 Amino acid sequences of analyzed flax proteins with lectin domains.

Supplementary File 2 Validation of RNA-Seq data by qRT-PCR analysis.

Supplementary File 3 RNA-Seq data for flax genes encoding proteins with lectin domains.

Supplementary File 4 Phylogenetic dendrogram of GNA (PF01453) lectin family members of *Arabidopsis thaliana* and *Linum usitatissimum*.
