# Supplementary material for: Gene Expression Patterns for Proteins With Lectin Domains in Flax Stem Tissues Are Related to Deposition of Distinct Cell Wall Types
Source: Front Plant Sci. 2021 Apr 26;12:634594. doi: 10.3389/fpls.2021.634594 (PMC8121149; doi:10.3389/fpls.2021.634594)
Supplement: Supplementary File 2 — Validation of RNA-Seq data by qRT-PCR analysis. [file Data_Sheet_2.pdf]

Supplementary File 2. Validation of RNA-Seq data by qRT-PCR analysis

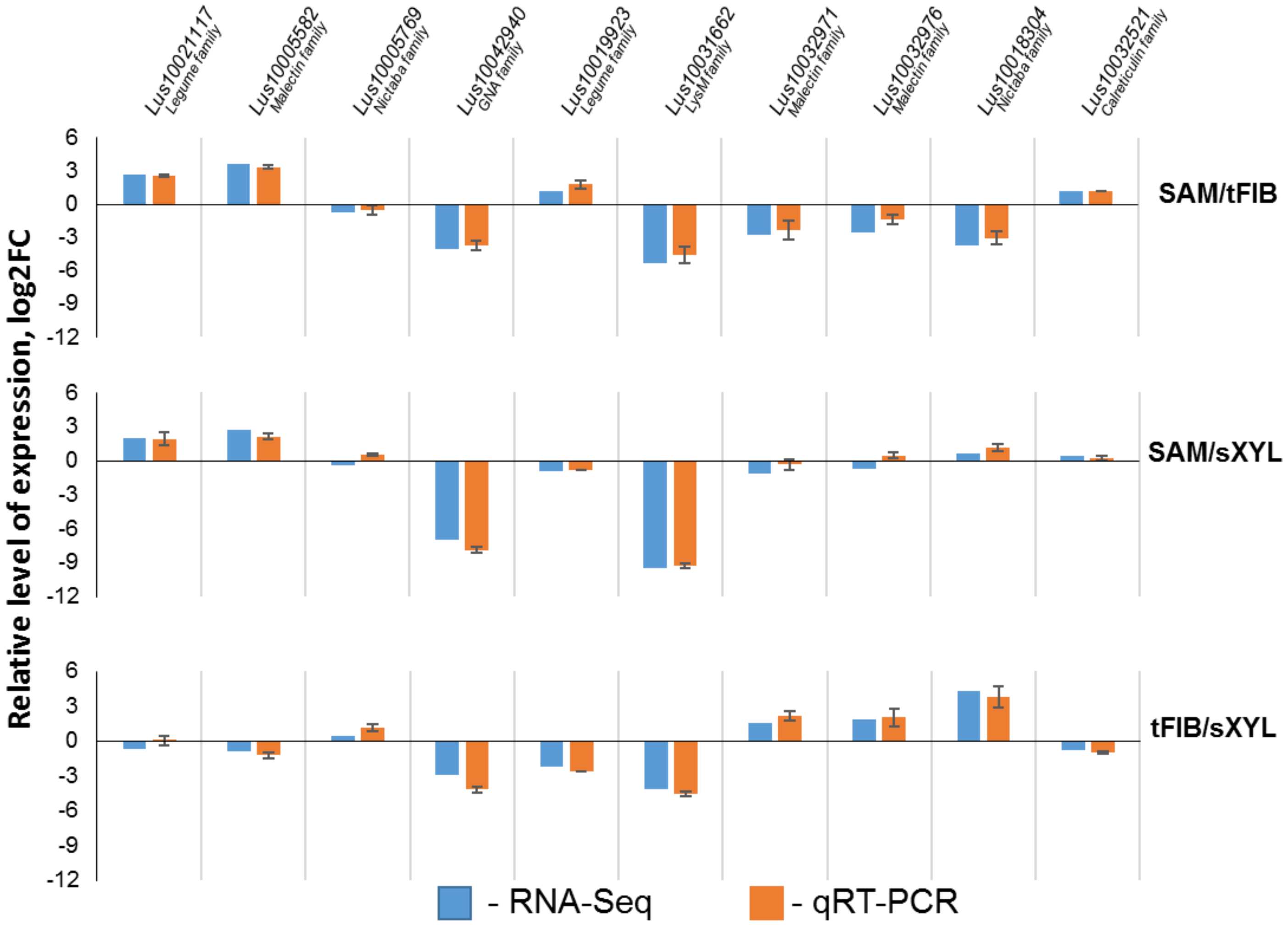

Figure S1. Comparison of gene expression values obtained by qRT-PCR and RNA-Seq analyses for 10 genes with various expression patterns. Given are the ratios of log_2FC of expression values obtained by qRT-PCR (orange columns) and RNA-Seq (blue columns) for apical stem region (SAM), xylem tissues (sXYL), and isolated phloem fibers (tFIB). Ratios of SAM/tFIB, SAM/sXYL and tFIB/sXYL samples were analyzed. Error bars indicate standard deviations. Similar ratios were obtained by both techniques validating the results of transcriptomic analysis.

To validate RNA-SEQ data real-time polymerase chain reaction (qRT-PCR) was carried out on a CFX96 TouchRT-PCR Detection System (Bio-Rad, Hercules, CA). mRNA from apical part of flax plants (2-3mm) that roughly corresponded to SAM samples (Zhang and Deyholos, 2016), as well as from tFIBa, tFIBb, sXYLa, and sXYLb samples was extracted using “ExtractRNA” (Evrogen, Moscow, Russia) and afterwards purified using an RNeasy Mini Kit (Qiagen, Germany). Obtained RNA samples were treated using a Turbo DNase Kit (Ambion, Austin, TX, USA). RNA concentration and integrity were tested with agarose gel electrophoresis and a NanodropND-1000 spectrophotometer (NanoDrop Technologies, Wilmington, DE, USA). 1 µg of RNA was used for cDNA synthesis using RevertAid H Minus Reverse Transcriptase (Thermo Fisher Scientific, Waltham, MA, USA) and Oligo (dT) 12-18 Primer (Invitrogen, Carlsbad, CA, USA) according to the manufacturer’s recommendations. 3 µl of 20-fold-diluted cDNA were used as the template for qPCR that was performed using a 2.5× Reaction Mix for qPCR with EVA Green dye (Syntol, Moscow, Russia) under the following conditions: 95ºC for 2 min, followed by 40 cycles at 94ºC for 10 s, 60ºC for 15 s and 72ºC for 30s. The final stage was the melt curve analysis in a temperature range of 60–95ºC. For validation of RNA-SEQ data, 10 genes with differed pattern of expression were chosen.

Sequences for gene-specific primers for the analyzed genes are presented in the Table S1 below. *GAPDH*, *ETIF1*, and *ETIF5A* genes were used as reference genes for qPCR data normalization (Huis et al., 2010). The amount of fluorescence was plotted as a function of the PCR cycle using CFX Manager Software (Bio-Rad, USA). The amplification efficiency for all primers was determined using a dilution series of a pool of cDNAs. Two technical repetitions were performed for each of three biological replicates.

The expression levels of target genes were calculated relative to the reference genes, and then the ratios of expression in different samples were calculated. Values of the relative level of gene expression for tFIBa and tFIBb, as well as for sXYLa and sXYLb were averaged and named as tFIB and sXYL, correspondingly (same as it was done for RNA-Seq data).

Table S1. List of genes chosen for validation and sequences of primers. pCW – primary cell wall, sCW– secondary cell wall, tCW – tertiary cell wall.

Gene ID (according to Phytozome)	PFAM/gene name	Lectin family	Specific expression	Primer_forward, 5'-3'	Primer_reverse, 5'-3'
Lus10021117	PF00139	Legume	pCW	TTCTTCAATCAACGCATT	CAATCTGACTTCTAACCTT
Lus10005582	PF11721	Malectin	pCW	AATTGTCTCCTGAGTCAT	CCACATTGATGGTTATCC
Lus10005769	PF14299	Nictaba	pCW	TTACAAAGTCCACGGATG	GTCAAGTAACGCTCAGAC
Lus10042940	PF01453	GNA	sCW	TCACTGCAGGTACCAACGAC	GCCGAGTGGGAAGAATCCAA
Lus10019923	PF00139	Legume	sCW	TAGTCCTGTCGCCGCTGCTTTC	CGGCGGCGACGCTTTGAC
Lus10031662	PF01476	LysM	sCW	GAAGGGGAATCAGCTCCTCG	TCGCTTATGGTGTTCAGCGT
Lus10032971	PF11721	Malectin	tCW	GCGGTGAAGGTGTGGAGTTA	TTTGAACCACCACTGCCACT
Lus10032976	PF11721	Malectin	tCW	CCGACGACGACGATCTGAAT	ATTTCTGGCCTGAGGTGACG
Lus10018304	PF14299	Nictaba	tCW	AGAAACAAAGTGAAACAAG	TGATCGGTTCATTAGTTG
Lus10032521	PF00262	Calreticulin	pronounced expression	CTCAAGCCCCGCAGCTACG	CGCCCTTCAAATGGCTCA
*Lus10011375**	GAPDH		Reference gene	GACCATCAAACAAGGACTGGA	TGCTGCTGGGAATGATGTT
*Lus10002264**	ETIF1		Reference gene	CTCAGGTGATGCGAATGCT	AATCCCTCAGCCCTACAAGG
*Lus10036801**	ETIF5A		Reference gene	CCGGAGCCTCCAAGACTTA	TGACGATGTATCCGTTCTTACG

* Huis, R., Hawkins, S., Neutelings, G. (2010). Selection of reference genes for quantitative gene expression normalization in flax (Linum usitatissimum L.). BMC Plant Biol., 10 (1), 1-14.
