# Supplementary material for: Gene Expression Patterns for Proteins With Lectin Domains in Flax Stem Tissues Are Related to Deposition of Distinct Cell Wall Types
Source: Front Plant Sci. 2021 Apr 26;12:634594. doi: 10.3389/fpls.2021.634594 (PMC8121149; doi:10.3389/fpls.2021.634594)
Supplement: Supplementary File 4 — Phylogenetic dendrogram of GNA (PF01453) lectin family members of Arabidopsis thaliana and Linum usitatissimum. [file Data_Sheet_4.pdf]

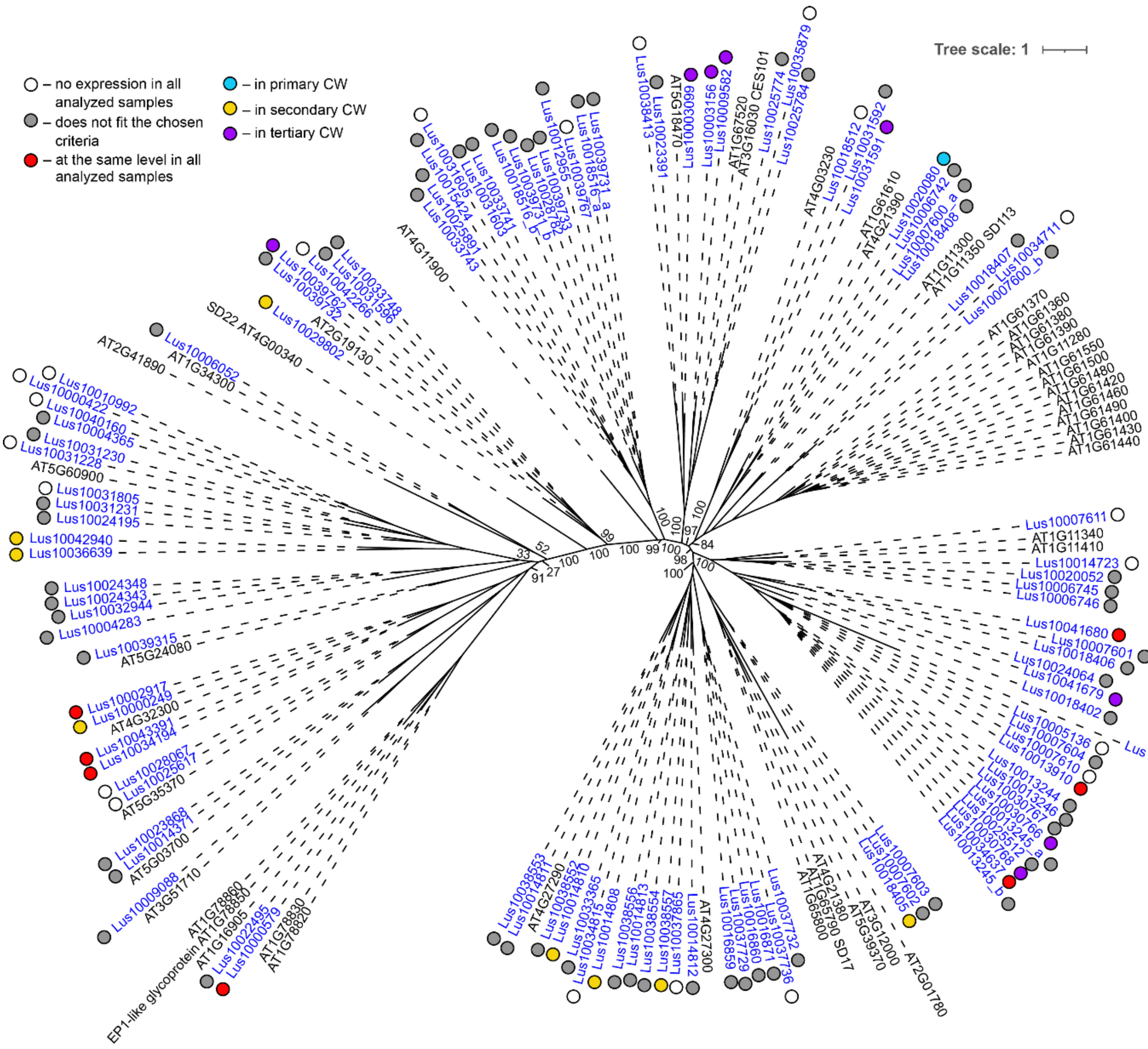

Supplementary File 4. Phylogenetic dendrogram of GNA (PF01453) lectin family members of *Arabidopsis thaliana* and *Linum usitatissimum*. The *A. thaliana* gene names are given in black font; additional gene names are given according to descriptions in the Uniprot database (https://www.uniprot.org/; Consortium, 2019). The *L. usitatissimum* gene names are given in dark blue font. The different colored dots next to *L. usitatissimum* gene names indicate genes with different expression patterns. The criteria used for different expression pattern identification as follow: red, the mean values of tau-score < 0.15 across all samples; light blue, DEGs upregulated in samples with primary CW; yellow, DEGs upregulated in samples with secondary CW; purple dots, DEGs upregulated in samples with tertiary CW. White dots indicate genes that are not expressed in the analyzed flax samples, and gray dots indicate genes where the expression does not fit the chosen criteria for a certain expression pattern (expression values for these genes are given in Supplementary File 2). The genes with red, light blue, yellow, and purple dots are listed in Tables 2, 3, 4 and 5, respectively. Numbers indicate the ultrafast bootstrap support values for some branches. Abbreviations: DEGs – differentially expressed genes, a pairwise comparison log2FC≥1, padj≥0.01, CW – cell wall.
